# High-current density alkaline electrolyzers: The role of Nafion binder content in the catalyst coatings and techno-economic analysis

**DOI:** 10.3389/fchem.2022.1045212

**Published:** 2022-10-28

**Authors:** Marilena Isabella Zappia, Sebastiano Bellani, Yong Zuo, Michele Ferri, Filippo Drago, Liberato Manna, Francesco Bonaccorso

**Affiliations:** ^1^ BeDimensional S.p.A, Genova, Italy; ^2^ Nanochemistry Department, Istituto Italiano di Tecnologia, Genova, Italy

**Keywords:** alkaline electrolyzers, hydrogen evolution reaction, oxygen evolution reaction, electrodes, nafion, platinum on vulcan (Pt/C)

## Abstract

We report high-current density operating alkaline (water) electrolyzers (AELs) based on platinum on Vulcan (Pt/C) cathodes and stainless-steel anodes. By optimizing the binder (Nafion ionomer) and Pt mass loading (m_Pt_) content in the catalysts coating at the cathode side, the AEL can operate at the following (current density, voltage, energy efficiency -based on the hydrogen higher heating value-) conditions (1.0 A cm^−2^, 1.68 V, 87.8%) (2.0 A cm^−2^, 1.85 V, 79.9%) (7.0 A cm^−2^, 2.38 V, 62.3%). The optimal amount of binder content (25 wt%) also ensures stable AEL performances, as proved through dedicated intermittent (ON-OFF) accelerated stress tests and continuous operation at 1 A cm^−2^, for which a nearly zero average voltage increase rate was measured over 335 h. The designed AELs can therefore reach proton-exchange membrane electrolyzer-like performance, without relying on the use of scarce anode catalysts, namely, iridium. Contrary to common opinions, our preliminary techno-economic analysis shows that the Pt/C cathode-enabled high-current density operation of single cell AELs can also reduce substantially the impact of capital expenditures (CAPEX) on the overall cost of the green hydrogen, leading CAPEX to operating expenses (OPEX) cost ratio <10% for single cell current densities ≥0.8 A cm^−2^. Thus, we estimate a hydrogen production cost as low as $2.06 kg_H2_^−1^ for a 30 years-lifetime 1 MW-scale AEL plant using Pt/C cathodes with m_Pt_ of 150 μg cm^−2^ and operating at single cell current densities of 0.6–0.8 A cm^−2^. Thus, Pt/C cathodes enable the realization of AELs that can efficiently operate at high current densities, leading to low OPEX while even benefiting the CAPEX due to their superior plant compactness compared to traditional AELs.

## Introduction

Energy storage through electrochemical production of green hydrogen is a crucial technology to empower the energy transition towards climate neutrality via grid integration of intermittent renewable energy sources and economy electrification. (Oliveira et al., 2021), (Mac Dowell et al., 2021) According to both national (U.S. and China)^1,2^ and international (Europe) technology roadmaps,^1^ hydrogen could account for more than 10–25% of the final country’s energy demand by 2050. This will balance the fluctuation of renewable energy sources by means of (inter-)seasonal storage, (Nicita et al., 2020), (Brauns and Turek, 2020) while permitting cost-effective emission-free transport of energy and people across regions, (Luo et al., 2020), (Sharma and Ghoshal, 2015) as well as industrial processes using high-grade heat (Oliveira et al., 2021). In this scenario, alkaline electrolyzer (AEL) stacks have been robustly established at MW-scale for over a century, with single-stack capacities up to several MWs. (Buttler and Spliethoff, 2018), (Brauns and Turek, 2020), (Krishnan et al., 2020) Meanwhile, research efforts have been devoted to increasing the energy efficiency of water electrolysis by screening alternative technologies to AELs, including proton-exchange membrane electrolyzers (PEMELs), (Ayers, 2021), solid oxide electrolyzers (SOELs), (Zheng et al., 2017), high-temperature alkaline electrolyzers (HTAELs), (Lohmann-Richters et al., 2021), anion-exchange membrane electrolyzers (AEMELs), (Vincent and Bessarabov, 2018), and proton-conducting ceramic electrolyzers (PCCELs) (Ding et al., 2020). Such AEL alternatives aim at competing against other forms of non-green hydrogen production via hydrocarbon fuel processing, (Nikolaidis and Poullikkas, 2017) *e.g*., steam methane reforming, (Iulianelli et al., 2016), partial oxidation (Sengodan et al., 2018) and coal gasification. (Midilli et al., 2021). In fact, “*AELs operate with satisfactory efficiency just at low current density”* is a commonly consented opinion. (Buttler and Spliethoff, 2018), (Bodner et al., 2015) This translates into green hydrogen production through bulky (non-compact) AEL plants that would require either high-capital expenditure (CAPEX) infrastructures, when operating at low current densities (<0.5 A cm^−2^), or excessive operating expenses (OPEX), when operating at high current densities (>0.5 A cm^−2^). Nevertheless, in our opinion, this common sense does not consider several advancements achieved in the design of highly performant electrocatalysts and diaphragms for AELs, which can lead to energy efficiency comparable to those of Pt-group metals (PGMs, *e.g*., Pt and Ir)-based PEMELs. (Schalenbach et al., 2016), (Lee et al., 2022) In addition, based on the average data of worldwide currently operating MW-scale AEL plants,^2^ and considering that their CAPEX are depreciated on the plant lifetime (10 + years, *e.g.*, 20–30 years)^3^, OPEX represent the most impacting costs for the green hydrogen productions for “long-living” AEL plants. This is especially true for AEL plants operating at high-current densities (*e.g*., ≥0.5 A cm^−2^, hydrogen production rate ≥0.186 kg m^−2^ s^−1^). Not by chance, Pt has been recently incorporated in the electrodes of zero-gap AEL prototypes to increase their performance (*i.e*., to decrease their OPEX), thus, aiming at lowering the cost outlook of green hydrogen.^4^ Importantly, the current Pt mine production is sufficient to satisfy a GW-scale of electrolyzer market. (Minke et al., 2021). By rationalizing these concepts, it is interesting to evaluate if the use of Pt-based cathodes in AELs can: 1) marginally impact on the CAPEX contribution to the overall hydrogen production cost by permitting durable high-current density operation like PEMELs; 2) minimize OPEX at high current densities (even higher than 1 A cm^−2^) by reaching PEMEL-like performances. In addition, such a type of Pt-based AELs will intrinsically avoid the use of PGMs-based anode catalysts such as those based on Ir_,_ (Minke et al., 2021), whose global mine production is currently insufficient to meet the demand for tens of GW-scale PEMEL market. (Minke et al., 2021). Nevertheless, as a preliminary step, it remains crucial to optimize Pt-based cathodes for AELs, for example by screening the optimal amount of both Pt, conductive additives (*e.g*., carbon materials) and binding agents (*e.g*., polymeric binders). In fact, while the optimization of the binder type and content is crucial for the design of catalyst coatings in electrode membrane assemblies (MEAs) of polymer electrolyte membrane electrolyzers (Mayerhöfer et al., 2021) (*i.e*., PEMELs (Xu and Scott, 2010), (Trinke et al., 2019) and AEMELs (Cho et al., 2018), (Cho et al., 2017a), (Masel et al., 2016), (Chen et al., 2021), (Li et al., 2020), (Plevová et al., 2022), (Koch et al., 2021)) or proton-exchange membrane fuel cells, (Jeon et al., 2010), (Cho et al., 2017b), (Antolini et al., 1999) this task has not been fully covered for AELs. Actually, in both PEMELs (Xu and Scott, 2010), (Bühler et al., 2019) and AEMELs, (Cho et al., 2018), (Cho et al., 2017a), (Masel et al., 2016) the incorporation of ionomer binders in the catalysts coating extends the ion conduction from the bulk of the membrane to the surface of the catalysts (guaranteeing the ion transport from the wet electrode (*i.e*., anode) to dried one (*i.e*., cathode). Differently, AEL electrodes (*e.g*., conventional Raney-type Ni electrodes (Gannon and Dunnill, 2019)) operate in “wet conditions”, (Najafi et al., 2022) meaning that the optimization of binder type and content may substantially differ from that of PEMELs and AEMELs. In general, the removal of gaseous hydrogen from the catalyst coating can be impeded when binder content exceeds a certain threshold, as a consequence of the decrease of the electrode porosity. (Plevová et al., 2022), (Koch et al., 2021) Such an effect may also lead to supersaturation of dissolved hydrogen, which in PEMELs has been identified as a cause of pronounced gas crossover losses. (Xu and Scott, 2010), (Trinke et al., 2019), (Bühler et al., 2019) Nevertheless, the incorporation of the binder should ensure long-term mechanical (and, thus, electrochemical) stability of the catalysts coating when it operates at high current density into practical AELs. (Cho et al., 2018), (Plevová et al., 2022), (Koch et al., 2021) In the latter, temperature as high as 80 °C and continuous liquid electrolyte circulation may also affect the electrode performances (Buttler and Spliethoff, 2018). Noteworthy, discrepancies between the catalysts performances measured in three-electrode cell configuration and electrolysis systems (including MEAs based on catalyst coated membranes -CCMs- or gas diffusion electrodes -GDEs-) have been recently discussed for both PEMEL and AEMELs, (Schröder et al., 2021), (Alinejad et al., 2020), (Ehelebe et al., 2022) but not for AELs, likely because they traditionally operate at current densities inferior to PEMELs and AEMELs, (Buttler and Spliethoff, 2018) *i.e*., not very far from those recorded in three-electrode cell setups. (Buttler and Spliethoff, 2018), (Chen et al., 2022), (Zayat et al., 2020) Therefore, the realization of high-current density AELs requires the elucidation of extra technical aspects that, until now, have been disregarded.

In this work, we report single cell AEL with state-of-the-art performance by optimizing a platinum on Vulcan (Pt/C)-based cathode formulation and using affordable anodes made of stainless-steel, calculating the resulting hydrogen production costs. In particular, the effect of the binder content and Pt mass loading (m_Pt_) of the cathode on the AEL performance is thoroughly analysed. To provide basic guidelines, we selected sulfonated tetrafluoroethylene-based fluoropolymer-copolymer (Nafion) as a prototypical ionomer binder to be used in our study. Nafion is the most used binder for the realization of Pt/C electrodes in PEMELs. Although Nafion is a proton-conducting ionomer, it is also widely used as a binder for Pt/C cathode in record-high performance AEMELs (Koshikawa et al., 2020). Nafion is also chemically stable under the harsh operating environment of AELs, *e.g*., 25–40% KOH electrolyte and temperature between 60–80°C, under which several anionic ionomers used for AEMEL electrodes undergo hydroxyl-induced degradation or suffer from CO_2_-induced formation of (bi)carbonates. (Abbasi et al., 2019), (Li D. et al., 2021), (Mustain et al., 2020) Without the need to recur to Ir-based anodes, the optimized single cell AELs based on stainless steel mesh (SSM) anodes reach durable PEMEL-like performances. A preliminary techno-economic analysis (TEA) is also carried out to highlight the potential of the as-developed Pt-based AELs. The hydrogen production cost, including both CAPEX and OPEX, is analysed as a function of m_Pt_ used in the Pt/C cathodes. By enabling the realization of AEL that can efficiently operate at high current densities, Pt cathode ensures low OPEX. Meanwhile, they potentially benefit the CAPEX because of the superior compactness of the resulting AEL plant compared to existing ones (at fixed net power). Considering the current Pt price, we propose the simple rule of thumb for our Pt/C cathode-enabled high-current density AELs: “*the higher the AEL performance, the lower the hydrogen production cost, regardless of the use of Pt needed for maximizing the cathode performances”.* Overall, we show the possibility to meet the worldwide (*e.g*., European Commission, China Hydrogen Alliance and U.S. Department of Energy) 2030 targets for the cost of green hydrogen (<$2.5 kg_H2_^−1^) (Kakoulaki et al., 2021), (Li Y. et al., 2021) with remarkable anticipation.

## Materials and methods

### Materials

Type 316 SSMs (90 × 90 mesh, 0.0035″ wire diameter), AvCarb MGL280 carbon papers (CPRs) (280 µm thickness), ELAT Hydrophilic Plain Carbon Cloth (406 microns thickness), D1021 Nafion™ Dispersion (10 wt%) were purchased from FuelCell Store. Zirfon Perl UTP 220 diaphragm was purchased from Agfa. Pt/C (C20-PT, 20 wt%) was supplied by QUINTECH. 2-propanol (IPA) (ACS reagent, ≥99.5%) was purchased from Sigma Aldrich.

### Electrode fabrication

The Pt/C cathodes were prepared through spray coating of inks of Pt/C in water:IPA (75:25), which were produced with a Pt/C concentration of 1 mg ml^−1^ and adding various amounts of Nafion dispersion (10 wt%) to get different weight contents of the ionomer binder in the final catalyst coatings, *i.e*., from 0 wt% to 80 wt% relatively to the solid content (*i.e*., Pt/C + Nafion). The inks were sonicated in an Ultrasonic Bath USC–THD (WVR) for 1 h to be homogeneously mixed. The so-produced inks were hand sprayed on CPRs mounted on a hot plate heated at 140°C, and the catalyst mass loading, *i.e*., m_Pt_, was controlled by adjusting the amount of the sprayed inks. For the measurements of electrochemically active surface area (ECSA) through hydrogen underpotential deposition (H_UPD_) measurements, rotating disk electrodes (RDE) were produced by depositing 20 µL of a Pt/C dispersion, prepared by adding 1 µL of Nafion dispersion (10 wt%) to 1 ml of a 1.3 mg ml^−1^ Pt/C dispersion, onto an RDE with a 5 mm diameter (mass_Pt_ ∼5.3 µg), which was dried at 60°C in air for 20 min.

### Electrode characterization

Inductively coupled plasma optical emission spectroscopy (ICP-OES) measurements were carried out on a ThermoFisher iCAP 7600 DUO Thermo spectrometer to measure m_Pt_ in the investigated cathodes. The samples were prepared by digesting a piece (0.57 cm^2^ area) of the electrode in 4 ml of HCl/HNO_3_ 3:1, v/v) for 18 h. The resulting solution was then diluted to 100 ml with Milli-Q water. The ICP measurements were affected by a systematic error of *ca.* 5%. Scanning electron microscopy (SEM) images were acquired on an FEI NanoLab 600 dual beam system with an acceleration voltage of 5–10 kV, while energy dispersive X-ray spectroscopy (EDS) was performed at the voltage of 20 kV. Electrochemical measurements of the cathodes were carried out using VMP3 Biologic potentiostat/galvanostat, equipped with an external high-current booster channel (Biologic). Galvanostatic polarization curves were acquired through multistep chronopotentiometry (CP) protocol using a three-electrode cell configuration in a polytetrafluoroethylene (PTFE) cell at room temperature. The cathode potential was measured over 5 min for each current step, and the final potential provided a point of the polarization curve. Potentiodynamic linear scan voltammetry (LSV) measurements were also performed with a 2 mV s^−1^ potential scan rate. A 6 M KOH-filled Hg/HgO electrode with a PTFE-body was used as the reference electrode, while a Pt wire was used as the counter electrode. The reference electrode was calibrated using standard calibration protocols (Niu et al., 2020). The electrolyte was 1 M KOH for both galvanostatic polarization curve and potentiodynamic LSV measurements. The galvanostatic polarization and potentiodynamic LSV curves were iR-corrected (i is the measured working electrode current and *R* is the series resistance) considering R as the resistance measured in high-frequency region through electrochemical impedance spectroscopy (EIS) measurements on the cathode and determined by the intercept of the Nyquist plot on the real impedance-axis. For the galvanostatic polarization curves, R was measured for each current step since the uncompensated resistance can change during measurements due to gas bubbles formation and temperature variation (Ehelebe et al., 2022). For the potentiodynamic LSV curves, R was measured at open circuit potential. Cyclic voltammetry (CV) measurements were acquired onto RDEs to measure the ECSA of Pt/C catalysts through the hydrogen underpotential deposition (H_UPD_) method. (Anantharaj et al., 2018), (Trasatti and Petrii, 1992) The potential scan rate was 50 mV s^−1^ and the 50th CV curve was analysed for the calculation of ECSA. The latter was determined by charge integration of the hydrogen adsorption region (Q_H_-_UPD_) in the CV curve after performing a double-layer current correction. (Anantharaj et al., 2018), (Trasatti and Petrii, 1992) Assuming a theoretical charge of 210 μC cm^−2^ for the absorption of a monolayer of hydrogen at the surface of polycrystalline Pt (Q_mono_), (Trasatti and Petrii, 1992), ECSA was calculated as: (Anantharaj et al., 2018), (Trasatti and Petrii, 1992)

$$ECSA=\frac{Q_{H-UPD}}{Q_{mono}\times {mass}_{Pt}}$$

### Alkaline electrolyzers assembly

The AELs were produced using a zero-gap single electrolysis cell (Dioxide Materials), including corrosion-resistant Ni-based anode and cathode flow field (bipolar) plates, o-ring seals, and Teflon gaskets. A piece of ELAT hydrophilic carbon cloth was used as extra gas diffusion layers (GDLs) at the cathode side. The cathode was ones of the investigated Pt/C cathodes, while stacked SSMs were used as anode in all the investigated AELs. Before use, the SSMs were cleaned with isopropanol/ethanol (1:1 vol./vol.) and distilled water and dried using an air stream. Zirfon Perl UTP 220 was used as diaphragm. The cell components were compressed during hardware assembling to realize the zero-gap single cell configurations. The AELs were connected to a custom-built station, which, through a peristaltic pump (Masterflex L/S Series), fed the anodic and cathodic half-cells with a 30 wt% KOH solution at a flow rate of 30 ml min^−1^ cm^−2^. The AELs operated at a temperature of 80°C (controlled with a proportional-integral derivative controller) and at an atmospheric (*ca.* 1 atm) system pressure.

### Alkaline electrolyzer characterization

The AELs operated with separate electrolyte cycles to avoid mixing of the anodic and cathodic electrolyte cycles of traditional AEL electrolysis, a practice recommended in previous reports (Trinke et al., 2018). This AEL operation management can limit the anodic hydrogen contamination, guaranteeing a safe operation without requiring extra measures (*e.g*., gas separating unit) to reduce the crossover or the hydrogen content within the anodic half-cell (Trinke et al., 2018). The electrolysis power was supplied to the AELs by a VMP3 Biologic potentiostat/galvanostat, equipped with an external high current booster channel. Galvanostatic polarization curves were acquired using a multistep CP protocol. The cell voltage was averaged over 3 min of each current step to provide a point of the polarization curve. To follow recommended practice guaranteeing the reproducibility of the polarization curves, (Karacan et al., 2022), the AELs were preconditioned recording 6 CV cycles between 1 V and 2 V at 5 mV s^−1^ voltage scan rate. The stability of the AELs was assessed by means of an accelerated stress test (AST). As similarly reported for other types of electrolyzers (*e.g*., PEM-ELs), (Morozan et al., 2020), the AST protocol involved AEL cycling between 1.00 A cm^−2^ (ON state) and 0.05 A cm^−2^ (OFF state), with each galvanostatic step kept for 15 min and a total test duration of 24 h. The stability of our most performant AEL was also evaluated in continuous mode though a CP protocol at a current density of 1 A cm^−2^ over 335 h. The voltage efficiency of the AELs was calculated assuming a Faradaic efficiency for the hydrogen evolution reaction (HER) equal to 1, thereby:

$$voltage\;efficiency=E_{rev}/E_{cell},$$

where E_cell_ is the cell voltage and E_rev_ is the thermodynamically reversible voltage for water electrolysis, *i.e*., the minimum voltage required for the onset of water electrolysis. In our AEL operating condition (temperature = 80°C, pressure = 1 atm) E_rev_ is 1.18 V. To facilitate the comparison of the performance of our AEL with those reported in literature, the following energy efficiency metrics are also calculated:

$$energy\;{efficiency}_{HHV}=\left(M_{H2}\times HHV\right)/{Energy}_{input}=E_{th}^{0}/E_{cell},$$

and

$$energy\;{efficiency}_{LHV}=\left(M_{H2}\times LHV\right)/{Energy}_{input}=1.25/E_{cell},$$

in which M_H2_ is the hydrogen gas weight produced by the AEL, HHV is the hydrogen higher heating value (141.7 kJ g_H2_^−1^), LHV is the hydrogen lower heating value (120.0 kJ g_H2_^−1^), E_th_^0^ (V) is the thermoneutral voltage for the water electrolysis at standard temperature and pressure conditions (*i.e*., 1.48 V), E_cell_, is the single cell voltage, Energy_input_ is the electric energy consumed to produce the hydrogen, calculated by multiplying the operating power of AEL by time. Though these efficiency metrics are commonly used in literature, Energy_input_ neglects some energy input contributions of the electrolyzer, such as the energy consumption from water peristaltic pumps and the thermal energy input (Lamy and Millet, 2020). Therefore, as discussed in ref. (Lamy and Millet, 2020), our energy efficiency metrics must be considered as approximated values to be used at laboratory level and for a straightforward comparison with literature results.

### Techno-economic analysis

A preliminary TEA was performed to estimate the CAPEX, OPEX and overall hydrogen production cost for an ideal 1 MW-scale AEL plant, assuming a complete performance retention from lab-scale tests to plant. The boundaries of the TEA were set at the outlet of the AEL, *i.e*., hydrogen stocking and transportation costs have not been considered.

The cost of the diaphragm/electrode package (DEP) for a single lab-scale cell (5 cm^2^) was calculated from the commercial price of each component or the price of its constituting raw materials (Supplementary Table S1). Manufacturing costs related to the cathodes (*i.e*., deposition of the catalytic coating by airbrushing) were not considered. Then, the CAPEX of an ideal 1 MW-scale AEL plant based on the DEP configurations tested at lab-scale was calculated starting from data provided by IRENA^5^ and reports on currently operating large-scale AEL plants (Lee et al., 2021) (Supplementary Table S2). In brief, the overall cost breakdown of a typical 1 + MW scale AEL has been used to retrieve the system CAPEX, including expenditures related to the Balance of Plant (BoP). The annual CAPEX was then calculated from overall CAPEX considering its depreciation through a capital recovery factor (CRF), as reported in the Supporting Information (Supplementary Table S3).

The OPEX of the plant was calculated starting from the data (*i.e*., current-voltage relationships) collected from our single cell AELs at lab-scale. Several entries were considered to sum up to the overall system OPEX, namely: electricity fed to the AEL, process water, labour, maintenance, and other ancillary costs (Supplementary Table S4). The total OPEX was computed summing up all the listed contributions and doubling the electricity-related expenses as BoP power consumption equals the AEL’s one for plant scales superior to 1 MW.

The amount of yearly produced H_2_ (kg_H2_ year^−1^) by the ideal AEL plant was calculated through the Faraday’s law:

$$annual\;H_{2}\;production=\frac{I\times t\times FE\times {MM}_{H2}}{n\times F}$$

where I is the total current delivered by the plant in 1 year, t is the time, FE is the Faradaic efficiency, MM_H2_ is the molecular mass of hydrogen (g mol^−1^), n is the number of electrons transferred for each H_2_ molecule generated (mol_e-_/mol_H2_) and F is the Faraday’s constant (C mol_e-_^−1^) (conversion factors not displayed).

Finally, the hydrogen production cost was calculated as:

$$H_{2}\;production\;cost\;\left(US\${/kg}_{H_{2}}\right)=\frac{Annual\;CAPEX+Annual\;OPEX}{Annual\;H_{2}\;production}$$

In addition, the best performing DEP configuration was subject to further analyses, investigating the effect of the single-cell current density and plant lifetime on the cost of produced hydrogen.

Further details on the assumptions made and parameters set throughout the TEA are available in the Supporting Information, which also features the Excel spreadsheet used to perform the analysis.

## Results

**FIGURE 1:**
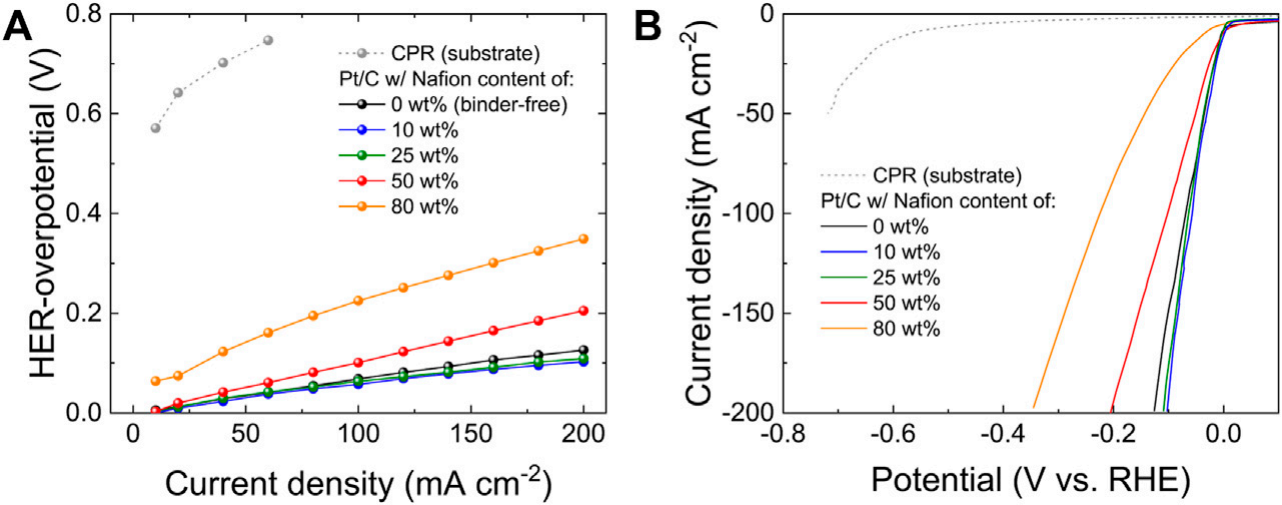
**(A)** Cathodic galvanostatic polarization curves and **(B)** potentiodynamic (polarization) LSV curves measured for the Pt/C electrodes produced with different Nafion contents in the catalyst coating, *i.e*., 0 wt% (binder-free electrode), 10 wt%, 25 wt%, 50 wt% and 80 wt%. The curves obtained for CPRs (electrode substrates) are also plotted.

We firstly investigated the effect of the Nafion (binder) content on the performances of Pt/C electrodes for the HER in alkaline media. The Pt/C catalysts used in this work are commercially available, with an ECSA of 39.5 m^2^ g^−1^, as estimated through the H_UPD_ method (Supplementary Figure S1). (Anantharaj et al., 2018), (Trasatti and Petrii, 1992) Considering the final implementation of the electrodes as cathodes in practical AELs, they were first designed using a constant m_Pt_ of ∼300 μg cm^−2^, which is on the same order of those recently reported in record-high performance AEMELs (Koshikawa et al., 2020), (Chen et al., 2021), (Masel et al., 2016), (Liu et al., 2017), (Cha et al., 2020), (Xiao et al., 2021), (Li et al., 2020) and common PEMELs (Bernt and Gasteiger, 2016), (Lewinski et al., 2015). The Nafion content in the Pt/C catalysts coating was varied between 0 wt% and 80 wt%. The polarization curves (Figure 1A) were acquired in galvanostatic mode (via a multistep CP protocol, see Materials and methods), following the best practices recommended for nanostructured electrodes. (Wei et al., 2019), (Anantharaj et al., 2021), (Anantharaj et al., 2018), (Voiry et al., 2018), (Anantharaj et al., 2022) In fact, potentiodynamic LSV measurements inevitably imply double-layer charging and other possible reactions, *e.g*., hydrogen adsorption, that lead to an inaccurate determination of the activity metrics. (Anantharaj et al., 2018), (Wei et al., 2019), (Anantharaj et al., 2021), (Voiry et al., 2018), (Anantharaj et al., 2022) Furthermore, we focused on the overpotential for the HER (*i.e*., absolute potential vs RHE) at 100 mA cm^−2^ (η_100_), instead of the one at 10 mA cm^−2^ (η_10_), as representative catalytic activity metric. This is key to avoid ambiguous interpretations arising from cathodic current originated by reduction of the oxygen functionalities of carbonaceous components (*i.e*., CPR and carbon black, namely Vulcan) in our high-mass loading electrodes, (Soliman et al., 2016). In fact, η_10_ strongly depends on side reactions beyond the HER, and such reactions scale with the electrode material mass loadings, (Anantharaj and Kundu, 2019), while η_100_ better reflects the final purpose of our work, which is the application of the designed Pt/C electrode as the cathode in AELs operating conditions. The data reveal that the most performant electrodes are those with Nafion contents of 10 wt% and 25 wt%, featuring η_100_ of 56 mV and 64 mV, respectively. By further increasing the Nafion content, the electrode catalytic activities deteriorate significantly, resulting in η_100_ of 101 mV and 225 mV for the Nafion contents of 50 wt% and 80 wt%, respectively. The binder-free electrode shows a satisfactory activity, *i.e*., η_100_ of 68 mV, approaching the most performant electrodes. However, catalyst detachment from the electrode surface was visible to the naked eye during the measurements. Potentiodynamic LSV curves (Figure 1B) were also acquired, confirming the activity trend obtained from the galvanostatic polarization curves. Being the initial m_Pt_ and the used Pt catalysts the same for all the electrodes, the mass activity (*i.e*., Pt mass-normalized cathodic current) and the ECSA-normalized specific activity reflect the same trend of the “geometric activity”, as expressed by the geometric current density.

**FIGURE 2:**
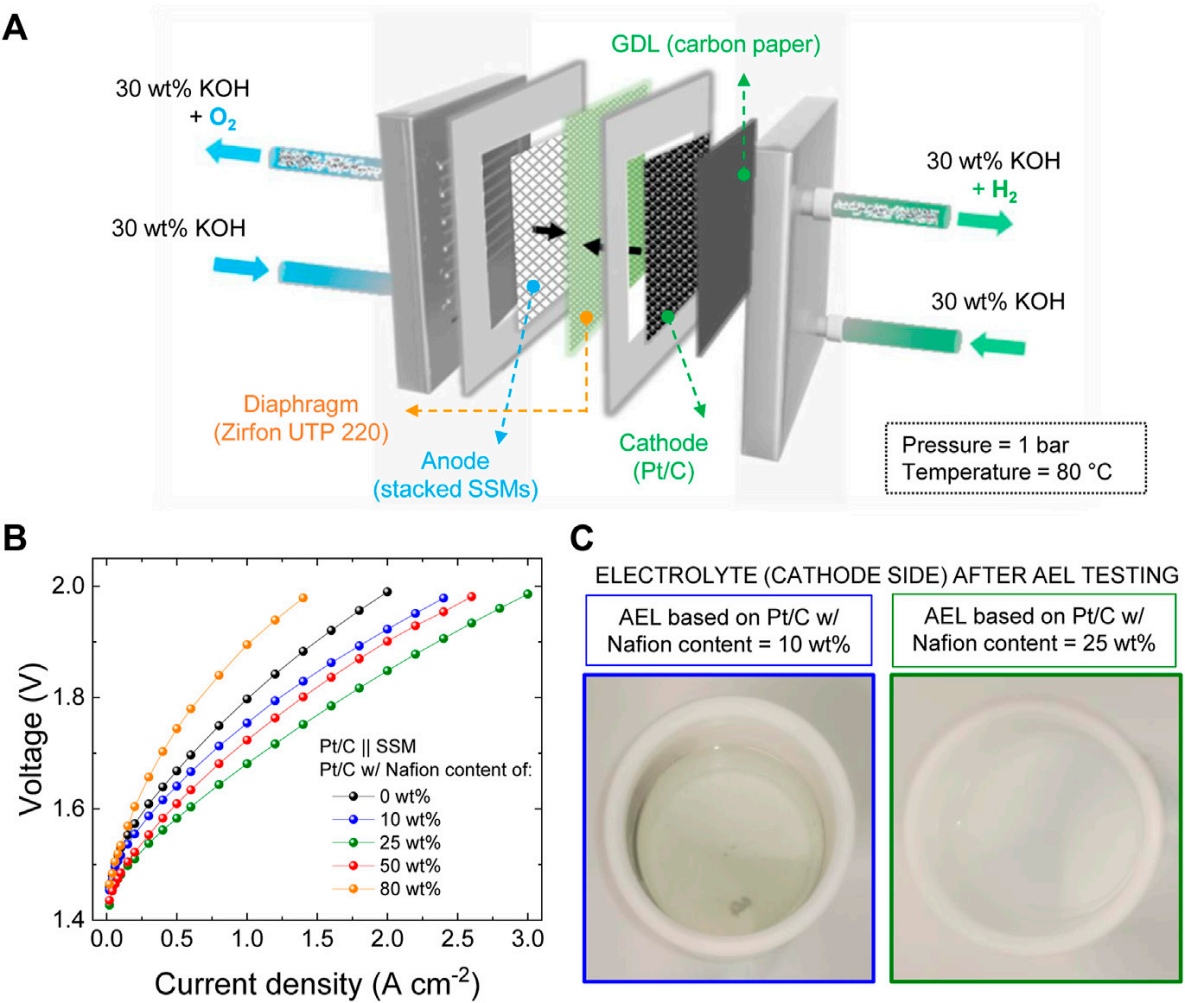
**(A)** Sketch of the configuration of our Pt/C || SSM AELs based on: a Pt/C cathode (+ CPR GDL), a SSM anode and a Zirfon UTP 220 diaphragm. Operating conditions: 30 wt% KOH electrolyte solution; atmospheric pressure (1 bar); 80 °C temperature; 30 ml min^−1^ flow rate. **(B)** Galvanostatic polarization curves measured for the AELs based on the Pt/C cathodes (m_Pt_ ∼300 μg cm^−2^) produced with different Nafion contents in the catalyst coatings, from 0 wt% (binder-free cathode) to 80 wt%. **(C)** Pictures of the post-test (10 CV cycles and polarization curve acquisition) electrolyte (cathode side) of the AELs based on the Pt/C cathode produced with a Nafion content of 10 wt% (left side) and 25 wt% (right side).

Although the three-electrode cell configuration tests enable a rapid assessment of the catalytic performance of an electrode, they do not implement the operating conditions of AEL electrodes. The latter operate at higher current densities (*e.g*., several hundreds of mA cm^−2^ or more), in more concentrated electrolytes (*e.g*, 30 wt% KOH or 20 wt% NaOH), while being subjected to additional mechanical stresses (*e.g*., cell torque, electrolyte circulation and pronounced gas bubbling) (Karacan et al., 2022). Thus, the main outcomes of the three-electrode cell configuration tests were cross-checked in single cell AELs. The latter were assembled by pairing our Pt/C electrodes (cathode) with stacked SSMs (anode) (Figure 2A), being stainless steel an inexpensive and robust catalyst for the oxygen evolution reaction (OER). (Chen et al., 2022), (Zayat et al., 2020), (Schäfer et al., 2018) Zirfon PERL UTP 220 membrane was used as diaphragm with high ionic conductivity (area-normalized ohmic resistance ∼0.1 Ω cm^2^) (Brauns et al., 2021) and low hydrogen crossover (anodic hydrogen content typically <2%, <0.2% at operating current density ≥500 mA cm^−2^, up to an operating pressure of 20 bar) (Brauns et al., 2021). Hereafter, the as-produced AELs are generically named Pt/C || SSM. Figure 2B shows the galvanostatic polarization curves of the atmospheric AELs, produced with the investigated Pt/C cathodes based on different Nafion contents, at 80°C and using 30 wt% KOH as the electrolyte solution. The Pt/C cathodes with 25 wt% Nafion content in the catalysts coating led to the best performance, corresponding to current densities of 0.5 A cm^−2^, 1.0 A cm^−2^ and 3.0 A cm^−2^ at 1.58 V, 1.68 V and 1.98 V, respectively. In accordance with the three-electrode cell configuration tests, excessive Nafion content in the Pt/C cathode (*i.e*., ≥50 wt%) led to performance deterioration (further explanation of this behaviour is reported hereafter considering the cathode morphology evaluated through SEM). However, insufficient Nafion content (*i.e*., ≤10 wt%) resulted in the detachment of the catalysts from the cathodes into the electrolyte solution, as indicated by the darkening of catholyte of the latter (Figure 2C **,** left side). Catalyst detachment was instead not observed for the most performant AEL using 25 wt% Nafion in the Pt/C cathode (Figure 2C, right side), suggesting that such binder content balances performance and stability.

**FIGURE 3:**
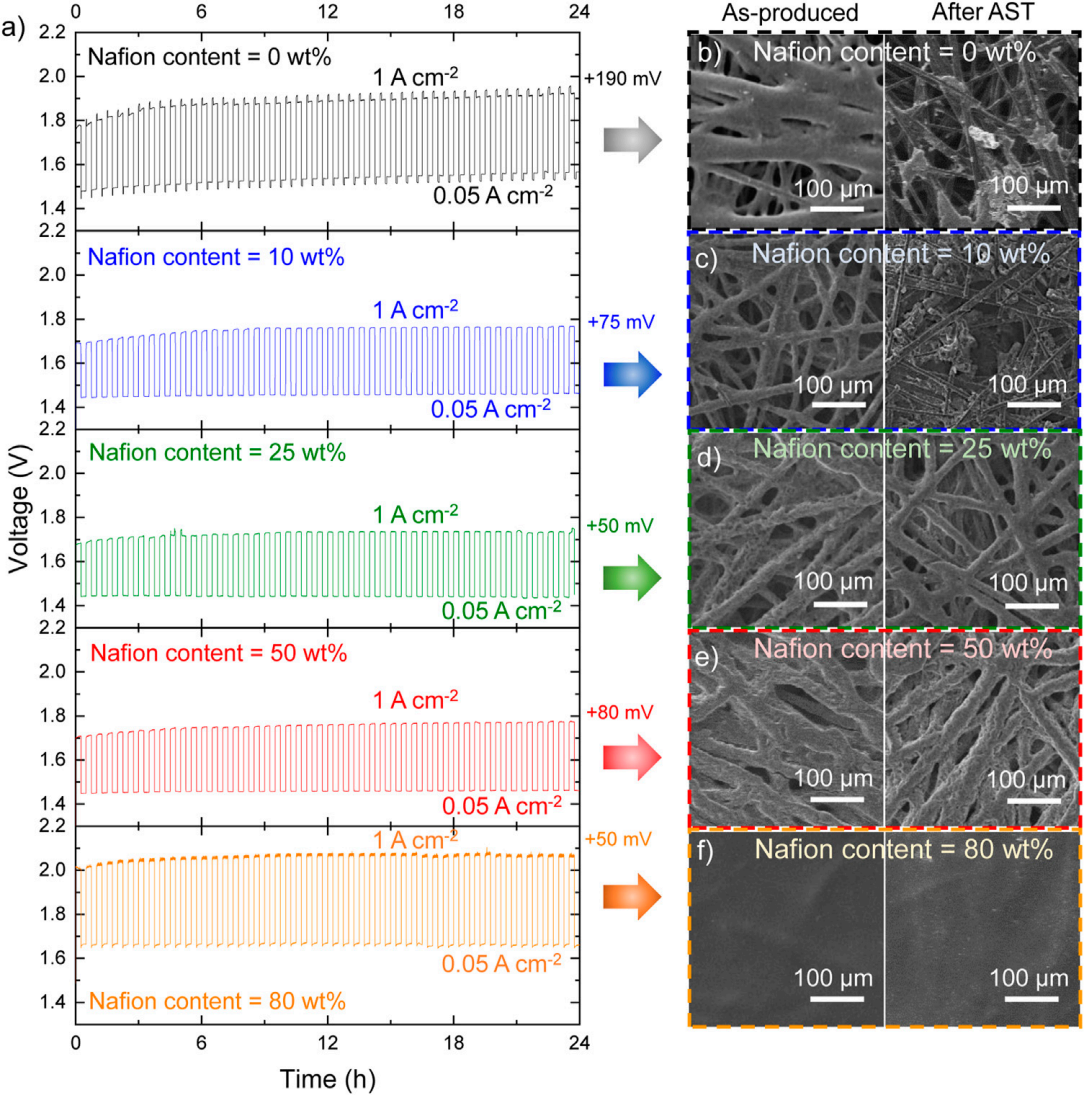
**(A)** AST tests for the Pt/C || SSM AELs based on Pt/C cathodes (m_Pt_ ∼300 μg cm^−2^) produced with different Nafion contents in the catalyst layer. The voltage increase at 1 A cm^−2^ after the AST is also indicated for each AEL. **(B–F)** SEM images of representative surface regions of the Pt/C cathodes before and after the ASTs of the corresponding AELs.

We carried out aging test to understand the impact of Nafion content on the durability of the AELs. The tests were carried out following a 24 h-AST procedure, consisting of cycling each AEL between 1.00 A cm^−2^ and 0.05 A cm^−2^, with each galvanostatic step kept for 15 min, over a full day. As shown in Figure 3A, the most and less performant AELs (Pt/C cathodes with 25 wt% and 80 wt% Nafion content, respectively) exhibited a nearly stable voltage at 1 A cm^−2^, leading to a +50 mV voltage increase at the end of the test. The AEL based on the Nafion-free Pt/C cathode was the most unstable device, with a +190 mV voltage increase at 1 A cm^−2^ at the end of the AST. Intermediate stabilities were observed for the other investigated AELs. These data first confirmed that the Nafion content must be optimized to attain the optimal trade-off between performance and stability of AELs using electrodes based on nanostructured catalysts coatings, as those of this work.

**FIGURE 4:**
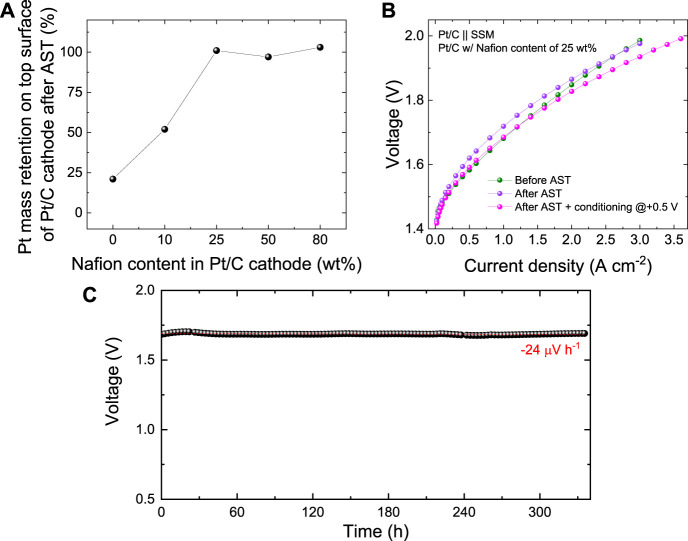
**(A)** Pt mass retention in the catalyst layer (top surface of the Pt/C cathode) after AST as a function of Nafion content in the catalysts layer. **(B)** Galvanostatic polarization curve of the AEL based on Pt/C produced with 25 wt% Nafion content in the catalysts layer, before and after tests, as well as after a further electrochemical conditioning of the AST-tested AEL. **(C)** Continuous stability test (CP protocol at 1.0 A cm^−2^) of the AEL based on Pt/C produced with 25 wt% Nafion content in the catalysts layer. The red dashed line is the linear fit of the data.

Scanning electron microscopy images were acquired on both pristine and used Pt/C cathodes to evaluate the changes of the surface morphology caused by the AST. In the as-produced Pt/C cathodes with Nafion contents from 0 wt% to 50 wt% (Figures 3B–E, left panels), the catalyst coating well covers the graphitic fibres of the CPR substrates while maintaining the macroporosity of the latter. Conversely, for the highest Nafion content of 80 wt%, the catalyst coating consists of a carpet-like compact surface layer (Figure 3F, left panel). It is reasonable to associate the blockage of the pores to a limited ECSA of the Pt/C cathodes. Noteworthy, our Pt/C cathodes have a m_Pt_ ∼300 μg cm^−2^. Consequently, their ECSA cannot be accurately measured by means of traditional RDE setup, where m_Pt_ of the investigated RDE is typically on the order of 10 μg cm^−2^. Even if the adsorption of side-chain sulfonate groups of Nafion on Pt surfaces has been demonstrated by spectroscopic studies (Gómez-Marín et al., 2010) or CO displacement experiments (Subbaraman et al., 2010), previous literature has shown that the Nafion has a negligible effect on the ECSA of Pt/C RDEs (Zhu et al., 2016). Thus, at this stage, we exclude an effect of adsorbed sulfonate groups on the catalytic activity of Pt in our cathode. Instead, the decrease of the porosity of the catalyst coating with increasing the Nafion content can explain well the reduced catalytic activity of Pt/C cathode with Nafion content higher than 25 wt%. In addition, in accordance with previous literature, (Trinke et al., 2019), excessive Nafion content (*e.g*., >25 wt%) can be also associated to a poor removal of evolved gaseous hydrogen, which may then insulate the catalytic sites from the electrolyte during the HER. After AST, the Pt/C cathodes with Nafion content ≥25 wt% did not show any significant morphology modification. Instead, the Pt/C cathodes with Nafion content ≤10 wt% revealed a substantial detachment of the catalyst coatings, uncovering the CPR graphitic fibres. This effect is consistent with the catalysts coating detachment, as assessed by the visual inspection of the cathode electrolyte (Figure 2C, left panel). The detachment of catalyst coatings for Nafion content ≤10 wt% was further confirmed by EDS analysis, showing the full retention of the Pt content in Pt/C cathodes with Nafion content ≥25 wt% (Figure 4A, see EDS maps measured for cathode surfaces before and after the AST in Supplementary Figures S2-S6). As recently shown in PEMELs, (Boulevard et al., 2022), catalysts coating damage can be also associated to the sudden onset of anomalous transient behaviour of voltage during the AST, *i.e*., the progressive occurrence of voltage spike after switching the current form 1.00 A cm^−2^ to 0.05 A cm^−2^ or vice versa (Figure 3A, top panel). A similar anomalous transient voltage behaviour (even if less intense) has been also observed for the AEL based on Pt/C cathode with 80 wt% Nafion content. In this case, no physical damage of the cathode was observed. Consequently, in this case, we speculate that the anomalous transient voltage behaviour is associated to mass transfer limitations caused by limited electrode porosity. No transient voltage behaviour anomalies were recorded for the AELs based on cathode with optimal Nafion contents (*e.g*., 25 wt%). To confirm the stability of our most performant Pt/C cathode during the AEL operation, the corresponding AEL was further evaluated through galvanostatic polarization curve after the AST. As shown in Figure 4B, the AEL still featured similar performances to those shown before AST for current density higher than 2.20 A cm^−2^. At lower current densities, the performance of the AEL slightly deteriorated after the AST test, which is consistent with the +50 mV increase observed at 1 A cm^−2^ at the end of the AST (see Figure 3A). Recent studies on AELs, as well as PEMELs and AEMELs, associated an initial (within the first 2 h) degradation of conventional AELs to the multiple effects, including: 1) catalysts detachment from electrodes; (Karacan et al., 2022), (Jin et al., 2021) 2) gas bubble coverage of the electrodes; (Karacan et al., 2022), (Chatzichristodoulou et al., 2016), (Zhang and Zeng, 2012), (Phillips et al., 2017) 3) modification of Ni components (*e.g*., catalysts, GDLs and bipolar plates). For instance, the absorption of atomic hydrogen in the nickel lattice leading to the formation of β-nickel hydride at the cathode side, (Rommal and Morgan, 1988), (Machado et al., 1994), (Soares et al., 1994) or the formation of NiO_2_, α/β -Ni(OH)_2_, and β-NiOOH at the anode side, (Karacan et al., 2022), (Hall et al., 2013) may cause interfacial resistance between the cell components (*e.g*., electrodes and bipolar plate) (Karacan et al., 2022). Our EDS analysis excluded catalysts detachment from electrodes. Moreover, the gas bubbling should impact more negatively with increasing the current density, and this was not observed. To consider the modification of Ni components, our AST-tested AEL was electrochemically conditioned at +0.5 V for 2 min, leading to a reverse current generically associated to [NiO_2_/β-NiOOH] [β NiOOH/Ni(OH)_2_] and [H_2_/H_2_O] redox couples, which, should accelerate the AEL performance degradation. (Uchino et al., 2018), (Todoroki and Wadayama, 2021) However, such electrochemical treatment restored the initial AEL performance for current densities equal to or lower than 1.5 A cm^−2^, while at higher current density the conditioned AEL resulted to be even more performant of the AEL than that before the AST test (Figure 4B). Beyond the above three effects, the electrochemical behaviour of SSM anodes should be also considered to explain the stability behaviour of our AELs. In fact, both dealloying and surface oxidation of SSM during constant-current electrolysis can result in hetero-layered Ni-Fe hydroxide/oxide nanostructures that show high OER-activity at high current density (*e.g*., 400 mA cm^−2^), even superior to that of state-of-the-art OER catalysts, *e.g*., IrO_2_ and Ni-Fe layered double hydroxides. (Todoroki and Wadayama, 2019). Such effect may already occur during the initial preconditioning of our AELs, consisting of 6 CV cycles between 1 V and 2 V at 5 mV s^−1^ voltage scan rate. (Karacan et al., 2022). Previous studies reported that prolonged OER under dynamic potential conditions can progressively densify the so-formed Ni-Fe hydroxide/oxide layers (Todoroki and Wadayama, 2021). In our AEL, this effect may be possibly associated to a decrease of the OER-activity of the SSM anode during AST test. By applying the “refreshing” electrochemical treatment, the Ni-Fe hydroxide/oxide layer can be reduced, while Cr and Fe dissolution can occur for anode potentials lower than their cathodic dissolution potentials (*i.e*., 0.73 and 1.13 V vs RHE respectively) (Todoroki and Wadayama, 2021). Nevertheless, focusing on the cathode side, our SEM-EDS analysis combined with electrochemical data indicates that our optimized Pt/C electrode (Nafion content = 25 wt%) are robust cathodes for high-performance AELs using cost-effective SSM anodes. To assess further the stability of our best AEL based on Pt/C cathode with a 25 wt% Nafion content, a continuous CP test was also performed by measuring the voltage of the AEL at 1.0 A cm^−2^ for 335 h. As shown in Figure 4C, the AEL showed a nearly ideal stability (nearly zero average voltage increase rate). A continuous AEL operation is therefore recommended to avoid electrochemical stresses, maximizing the overall lifetime of the AELs, thus, meeting the lifetime specification expressed by traditional AEL systems (≥10 years).

**FIGURE 5:**
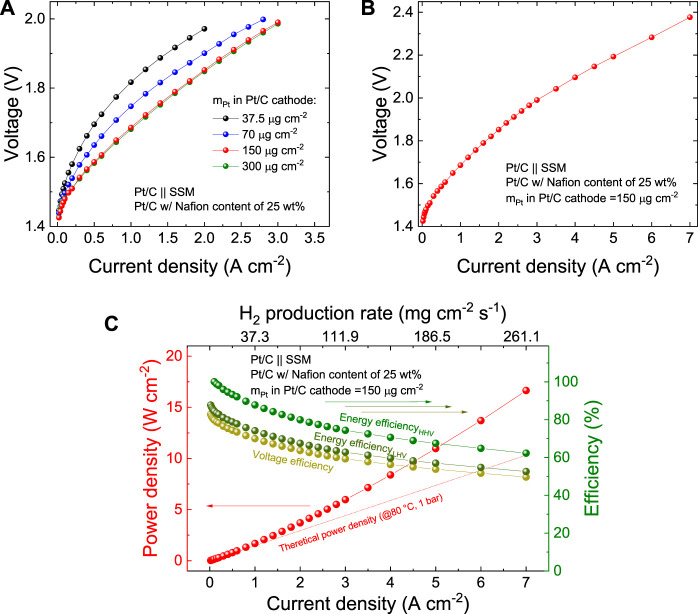
**(A)** Galvanostatic polarization curve of the AEL based on Pt/C cathodes produced with 25 wt% Nafion content in the catalysts layer and different m_Pt_ (from 37.5 to 300.0 μg cm^−2^). **(B)** Galvanostatic polarization curve, **(C)** power density and efficiency metric (*i.e*., voltage efficiency, energy efficiency_HHV_ and energy efficiency_LHV_) as a function of the current density of the AEL based on the Pt/C cathode produced with 25 wt% Nafion content in the catalysts layer and m_Pt_ of 150 μg cm^−2^, tested up to a current density of 7.0 A cm^−2^.

Once assessed the best Nafion content in our Pt/C cathodes, we evaluated the effect of the m_Pt_ of the Pt/C cathode on the AEL performance, aiming at proposing a competitive AEL technology in terms of hydrogen production cost finding an optimum between OPEX and CAPEX. Figure 5A shows that the AEL performances increase with m_Pt_ up to 150 μg cm^−2^. By further increasing m_Pt_ to 300 μg cm^−2^, the AEL performances remain very similar, suggesting that other limiting factors come into play (*e.g*., anode kinetics and ohmic resistance of diaphragm/electrolyte). As shown in Figure 5B, the AEL based on a Pt/C cathode with a m_Pt_ of 150 μg cm^−2^ was tested up to current density of 7 A cm^−2^, corresponding to a voltage of 2.38 V. Figure 5C plots the power density and efficiency of this AEL configuration as a function of the current density (or hydrogen production rate). The AEL operated with an energy efficiency_HHV_ of 93.3%, 87.8% 79.9%, 74.4%, 67.5%, 62.3% at 0.5, 1.0, 2.0, 3.0, 5.0 and 7.0 A cm^−2^, respectively. The performance of the optimized AELs are significantly superior to those of single cell in commercial AEL stacks, (Buttler and Spliethoff, 2018), and, to the best of our knowledge, represent the state of the art for AELs (Supplementary Table S5). Furthermore, the performance of the designed AELs approaches those achieved by PEMELs and AEMELs (see Supplementary Table S5), (Buttler and Spliethoff, 2018) but without relying on expensive Ir- (or Ru)-based anodes (Buttler and Spliethoff, 2018). Supplementary Figure S7 reports the galvanostatic polarization curves measured for a commercially available AEL stack and the corresponding single cell, showing that this cell operates at 0.5 A cm^−2^ with voltage of 1.79 V, which is 0.21 V higher than the one of our AEL at the same current density (1.58 V, see Figure 2B and Figure 4B).

**FIGURE 6:**
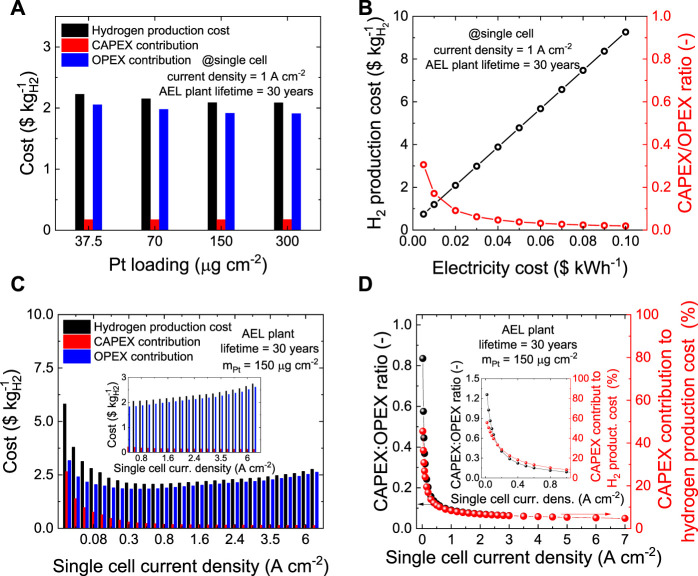
**(A)** OPEX, CAPEX and overall hydrogen production cost for ideal 1 MW-scale AEL plants based on the Pt/C cathodes with different m_Pt_ (single cell current density = 1 A cm^−2^; AEL plant lifetime = 30 years). **(B)** Hydrogen production cost and CAPEX/OPEX ratio for the 1 MW-scale AEL plant based on the Pt/C cathodes with m_Pt_ = 150 μg cm^−2^, as a function of the electricity cost (single cell current density = 1 A cm^−2^, AEL plant lifetime = 30 years). **(C)** OPEX, CAPEX and overall hydrogen production for 1 MW-scale AEL plant based on the Pt/C cathodes with m_Pt_ of 150 μg cm^−2^, as a function of single cell current density (AEL plant lifetime = 30 years). The inset panel shows a magnification of the high current density region. **(D)** CAPEX:OPEX ratio (left *y*-axis) and CAPEX contribution to hydrogen production costs (right *y*-axis) as a function of the single cell current density. The inset panel highlights the low current density region (≤1 A cm^−2^).

Figure 6A shows the impact of m_Pt_ in the Pt/C cathode on the CAPEX, OPEX and, overall hydrogen production costs as estimated by the TEA for an ideal 30 years-lifetime 1 MW-scale AEL plant operating at single cell current density of 1 A cm^−2^ (see additional details in Supplementary Tables S1–S4). In this case, m_Pt_ marginally impact on the CAPEX, which increases for less than $0.01 kg_H2_^−1^ for m_Pt_ increasing from 37.5 to 300.0 μg cm^−2^. Consequently, the hydrogen production costs are mainly determined by OPEX, which, in turn, depends on the AEL performance. Thus, the most profitable hydrogen production cost of ∼$2.09 kg_H2_^−1^ is obtained for both m_pt_ of 150 and 300 μg cm^−2^, as their similar performance yields the lowest OPEX (∼$1.91 kg_H2_^−1^ for both the cases) among the Pt loadings under study. The less profitable hydrogen production cost of ∼$2.23 kg_H2_^−1^ is instead obtained for m_Pt_ of 37.5 μg cm^−2^, driven to such value by the raised OPEX (∼$2.05 kg_H2_^−1^).

Despite these results, the data indicate that other factors beyond m_Pt_ and AEL performance are determining hydrogen production cost. As shown in Figure 6B, the electricity cost (assumed equal to $0.02 kWh^−1^ for data in Figure 6A, as envisaged by IRENA report^6^) has a great impact on the hydrogen production cost. By halving the electricity cost from $0.02 kWh^−1^ to $0.01 kWh^−1^, a minimum production cost of ∼$1.19 kg_H2_^−1^ is obtained for the best cases (expressed by m_Pt_ of 150 and 300 μg cm^−2^). Similarly, doubling the electricity cost results in the most profitable hydrogen production cost of $3.88 kg_H2_^−1^, while the worst case (m_Pt_ = 37.5 μg cm^−2^) yields $4.16 kg_H2_^−1^. The results of this sensitivity analysis depicts, once more, a picture in which the optimization of the catalytic system plays a marginal role on the overall hydrogen production cost when compared to the cost of energy.^4,6^ Nevertheless, green hydrogen production through electrolysis cannot refrain from an improvement of the electrolyzer performance to achieve profitability. In this view, it is important to notice that current commercial AELs do not operate at current densities as high as those considered above (*i.e*., 1 A cm^−2^). This is because of their insufficient energy efficiencies, which, in turn, increase substantially the OPEX, changing the cost trends here observed (Buttler and Spliethoff, 2018).

Even though it is not the purpose of this work to extrapolate a reliable TEA on commercial AEL technologies, a sensitivity analysis on the operative current-voltage of the ideal 1 MW-scale electrolyzer based on the best performing AEL configuration studied in this paper (*i.e*., m_Pt_ = 150 μg cm^−2^) was carried out. The impact of the single cell current density on the CAPEX, OPEX and hydrogen production cost is revealed in Figure 6C. The most profitable hydrogen production cost of $2.06 kg_H2_^−1^ is obtained for single cell current density between 0.6 and 0.8 A cm^−2^. Contrary to commercial AEL technologies, our AELs maximize their profitability when operate at single cell current density >0.5 A cm^−2^. This is a direct consequence of the high Pt/C activity for the HER, which, in turn, contain the OPEX in a broad range of operative conditions (OPEX < ∼$2.70 kg_H2_^−1^ for 7.0 A cm^−2^). As shown in Figure 6D, the single cell current density has a great impact on the CAPEX:OPEX ratio, showing that low single cell current densities significantly increase the contribution of CAPEX to the overall hydrogen production cost. Thus, for single cell current densities ≤0.1 A cm^−2^, CAPEX represents more than 20% of the hydrogen production costs. For current density ≥0.6 A cm^−2^, CAPEX is instead ≤10% of the hydrogen production costs. Thus, our results indicates that efficient high-current density AELs, here enabled using optimized Pt/C cathodes, may potentially reduce the CAPEX impact on the final hydrogen production cost as compared to traditional AELs (at fixed net power of the electrolyzer) because of the highest compactness of AEL plant dimension. Contrary to traditional AELs, (Buttler and Spliethoff, 2018), ours keep low OPEX at current density higher than 0.4 A cm^−2^, *e.g*., less than $2.70 kg_H2_^−1^ even at the highest single cell current density of 7 A cm^−2^. The latter depends on both the AEL performance (and thus, for our AELs, on m_Pt_, see Figure 6A) and the electricity cost (see Figure 6B). Finally, despite showing a remarkable stability to AST, our cathodes and anodes cannot be light-heartedly supposed to perform for periods as long as 30 years. Therefore, the competitiveness of short-living ideal 1 MW-scale AELs based on cathodes with m_Pt_ = 150 μg cm^−2^ has been investigated through a sensitivity analysis. The data obtained for AEL plant lifetime of 10 and 20 years are shown in Supplementary Figure S8, revealing that for the shortest lifetime (*i.e*., 10 years) the most profitable hydrogen production cost of $2.27 kg_H2_^−1^ is obtained at single cell current density between 0.8 and 1.0 A cm^−2^. Noteworthy, the overall CAPEX depreciation in short-living AEL plants results in the highest annual CAPEX. Consequently, the most profitable hydrogen production condition corresponds to single cell current densities higher than long-living AEL plants. Nevertheless, our experimental and TEA outcomes indicate that the worldwide (*e.g*., European Commission, China Hydrogen Alliance and U.S. Department of Energy) 2030 targets for the cost of green hydrogen (<$2.50 kg_H2_^−1^, at GW-market scale) (Kakoulaki et al., 2021), (Li Y. et al., 2021) can be met with severe anticipation by the proposed Pt/C-based AELs.

## Conclusion

In summary, we produced high-current density AELs based on Pt/C cathode and stainless-steel anodes. Firstly, the Nafion binder content in the catalyst coating of the Pt/C cathode was optimized, showing that a 25 wt% Nafion content results in optimal AEL performances and an approximately ideal stability (*i.e*., nearly zero average voltage increase rate). Thus, we revealed that the binder content optimization is a crucial aspect also in AELs using electrode based on nanostructured catalysts coatings (like GDE used in PEMEL and AEMELs), and not only in other water electrolyzer technology, such as PEMELs and AEMELs. Subsequently, the impact of m_Pt_ of the Pt/C cathode on the overall AEL performance was also investigated. At the optimal m_Pt_ of 150 μg cm^−2^, the AEL operated with the following (current density, voltage, energy efficiency_HHV_) conditions (1.0 A cm^−2^, 1.68 V, 87.8%) (2.0 A cm^−2^, 1.85 V, 79.9%) (7.0 A cm^−2^, 2.38 V, 62.3%). The performances of our AELs reached those of the most efficient PEMELs and AEMELs, and, despite the simplicity of our cell architecture, represent the state-of-the-art of AELs (to the best of our knowledge). Contrary to competing technologies, such as PEMELs and state-of-the-art AEMELs, the proposed system does not rely on expensive anodes based on Ir, whose global mine production may not be sufficient to enable future tens of GW-scale electrolyzer market. To prove the economic competitiveness of the developed AEL technology and the impact of m_Pt_, a preliminary TEA was performed for an ideal 1 MW-scale AEL plant implementing the herein investigated Pt/C cathodes. Our results show that, at single cell current density of 1 A cm^−2^, the m_Pt_ here investigated (from 37.5 to 300.0 μg cm^−2^) marginally impact on the CAPEX. Meanwhile, m_Pt_ of 150 μg cm^−2^ represents an estimate threshold at which the AEL is maximized (thus OPEX are minimized). More importantly, the CAPEX start to contribute significantly to the overall hydrogen production costs for single cell current densities lower than those used in commercial AELs (Buttler and Spliethoff, 2018) (>10% of the hydrogen cost for single cell current densities ≤0.1 A cm^−2^). Overall, the use of Pt/C cathodes enables efficient high-current density operation, leading to hydrogen production costs approaching $2.06 kg_H2_^−1^, as mainly determined by the single cell performance (and thus m_Pt_) and the electricity cost (here assumed equal to $0.02 kWh^−1^). Importantly, the proposed Pt/C cathode-based AEL technology has the potential to meet the worldwide 2030 targets for the hydrogen production cost with severe anticipation, in agreement with recent forecasts of electrolyzer manufactures located in South Africa, one of the main mineral resources of Pt.

## Data Availability

The original contributions presented in the study are included in the article/Supplementary Material, further inquiries can be directed to the corresponding authors.

## Footnotes

1 U.S. Department of Energy Hydrogen Program Plan, https://www.hydrogen.energy.gov/pdfs/hydrogen-program-plan-2020.pdf.

2 Energy in China’s New Era, the State Council Information Office of the People’s Republic of China, http://www.scio.gov.cn/zfbps/32832/Document/1695135/1695135.htm.

3 Hydrogen Roadmap Europe: A sustainable pathway for the European Energy Transition, https://www.fch.europa.eu/sites/default/files/Hydrogen%20Roadmap%20Europe_Report.pdf.

4 Green Hydrogen Cost Reduction—Scaling up electrolysers to meet the 1.5 °C climate goal. Int. Renew. Energy Agency (IRENA), https//irena.org/publications/2020/Dec/Green-hydrogen-cost-reduction.

5
https://www.miningweekly.com/article/pgms-playing-atypical-role-in-alkaline-electrolyser-lowering-green-hydrogen-cost-outlook-2021-10-26.

6 Green Hydrogen Cost Reduction—Scaling up electrolysers to meet the 1.5 °C climate goal. Int. Renew. Energy Agency (IRENA), 2020. https//irena.org/publications/2020/Dec/Green-hydrogen-cost-reduction.
