# Supplementary material for: High-current density alkaline electrolyzers: The role of Nafion binder content in the catalyst coatings and techno-economic analysis
Source: Front Chem. 2022 Oct 28;10:1045212. doi: 10.3389/fchem.2022.1045212 (PMC9649444; doi:10.3389/fchem.2022.1045212)
Supplement: Supplementary file 1 [file DataSheet1.pdf]

Supplementary Material

High-current density alkaline electrolyzers: the role of Nafion binder content in the catalyst coatings and technoeconomic analysis

Marilena Isabella Zappia,[1‡] Sebastiano Bellani,[1,‡*] Yong Zuo,[2] Michele Ferri,[2] Filippo Drago,[2] Liberato Manna,[2] Francesco Bonaccorso[1,*]

[1] BeDimensional S.p.A, Via Lungotorrente Secca, 30R, 16163 Genova, Italy

[2] Nanochemistry Department, Istituto Italiano di Tecnologia, Via Morego 30, 16163 Genova, Italy

‡ These authors equally contributed

* **Correspondence:** Sebastiano Bellani, Francesco Bonaccorso s.bellani@bedimensional.it; f.bonaccorso@bedimensional.it

Supplementary details on techno-economic (TEA) analysis

In the following, all the calculations and assumption made in the annexed Excel Spreadsheet are sheet-by-sheet described and commented. All parameters fixed and assumptions made are gathered in **Tables S1-4**.

"Description": reports the rationale and boundaries of the TEA.

"Single cell": reports the calculation of the unitary (\$ per 1 cm^2) and 5 cm^2-area lab-scale cell cost of the main components of the diaphragm/electrode package (DEP), namely cathode, anode and diaphragm.

Cathode: comprises the active phase, *i.e.*, Pt, a carbon-based support, *i.e.*, AvCarb MGL280, and a polymeric binder, *i.e.*, Nafion (see Materials and Methods in the main text). The unitary costs of each raw material included in the manufacture of the cathode was retrieved from a National Renewable Energy Laboratory (NREL) technical report,(Mayyas et al., 2019) while the exact Pt loading was determined by Inductively coupled plasma - optical emission spectrometry (ICP-OES), as described in the main text.

Anode: constituted by 5 stacked Type 316 stainless steel mesh (SSM) pieces, each one sized 5 cm^2. The SSM price from stainless-steel price.

Diaphragm: constituted by Zirfon PERL UTP 220. Being Zirfon PERL UTP 220 a commercial product, its price was retrieved from the supplier.

"CAPEX - Upscale to 1 MW": reports the calculations of the capital expenses (CAPEX) of the ideal 1 MW-scale AEL plant based on the DEP configurations studied in the present work. At first, the CAPEX of a generic 1 MW-scale AEL plant was estimated from literature data.[1],(Lee et al., 2021) Then, following the cost breakdown of a generic MW-scale AEL plant reported by IRENA[1] and assuming AEL performances to meet those reported by the Korean Institute of Energy Research (Lee et al., 2021), the CAPEX was split into stack, DEP and Balance of Plant (BoP) costs and relative subcomponents.

Finally, upscaling the size of the DEP (cathode, anode and diaphragm) to 700 cm^2 and assuming the final ideal AEL to be composed by 5 stacks of 200 unit cells,(Lee et al., 2021) the total CAPEX of an ideal 1 MW-scale AEL plant for each DEP configuration tested in the present work was calculated. Such CAPEX estimation was made under the assumption that cathode and anode costs scale linearly with plant size.

The annual CAPEX was retrieved from overall CAPEX considering its depreciation through a capital recovery factor (CRF), calculated according to the following equation:

$$\mathrm{CRF} = \frac{\mathrm{i_{Rate}} \times (1 + \mathrm{i_{Rate}})^{n}}{(1 + \mathrm{i_{rate}})^{n} - 1}$$

[1] Green Hydrogen Cost Reduction – Scaling up electrolysers to meet the 1.5 °C climate goal. Int. Renew. Energy Agency (IRENA), https//irena.org/publications/2020/Dec/Green-hydrogen-cost-reduction.

where i_{rate} is the discount rate and n is the AEL plant lifetime.

"OPEX - Upscale to 1 MW": reports the electrochemical data (*i.e.*, current-voltage relationships) collected with different DEP configurations on a 5 cm^2 AEL single cell operating under industrially relevant conditions. Also, energy efficiency based on hydrogen higher heating value (energy $efficiency_{HHV}$) and gross system power of the ideal 1 MW-scale AEL are shown for each configuration.

Major contributors of operating expenses (OPEX), *i.e.*, the electricity fed to the electrolyzer, the process water consumed, labor, maintenance and ancillary costs, were calculated. The OPEX related to electricity and process water, which are dependent on the electrolyzer performance, were calculated according to the gross power of the system and the water annual consumption, respectively.

In details, the cost of the electricity required for the actual electrolysis process was calculated according to the following equations:

$$I_{Total} = i \times A_{Single\ cell-Ideal\ 1\ MW-scale\ AEL} \times n_{Cells\ per\ stack} \times n_{Stacks\ per\ system}$$

$$P_{AEL\ (gross)} = I_{Tot} \times E_{Cell} \times t_{Annual\ AEL\ operation}$$

$$OPEX_{Electricity} = P_{AEL\ (gross)} \times C_{Electricity}$$

where I (i) indicates the current (current density) flowing through the electrolyzer, A stands for area, E for voltage, P for power, t for time and C for cost.

The expenses related to process water consumption have been estimated through the following equations:

$$m_{H2O\ consumed\ (per\ year)} = m_{Produced\ H2\ (per\ year)} \times m_{Average\ H2O\ consumption\ per\ kg\ of\ H2}$$

$$OPEX_{H2O} = m_{H2O\ consumed\ (per\ year)} \times C_{H2O}$$

where m stands for mass.

On the other hand, labor, maintenance and other ancillary OPEX contributions were calculated as percentages of the total CAPEX of the whole system.

"Annual H_2 productivity at 1 MW": reports the amount of yearly produced hydrogen for each DEP configuration, all up-scaled to a 1 MW scale. Refer to the dedicated paragraph in the Experimental Section for details.

"Sensitivity to electricity cost": reports the variation of the overall H_2 production cost, CAPEX and OPEX contribution to the latter and CAPEX/OPEX ratio for different costs of the electricity used to power the AEL. Further details are available in the main text and in the Excel Spreadsheet.

"H_2 production cost vs Pt loading": reports the final calculation of the production cost of H_2, starting from CAPEX and OPEX values and H_2 annual productivity for each DEP configuration.

According to reports on currently operative AELs,[1] the energy consumption for the actual electrolytic process accounts only for the 50% of the overall energy fed to the whole system, with BoP auxiliaries (gas and liquid circulation, gas compression…) requiring a similar energy fed. Therefore, the annual OPEX has been calculated doubling the $OPEX_{Electricity}$. Refer to the dedicated paragraph in the Experimental Section for details.

"H_2 cost vs I, best Pt loading 10 – 20 – 30": reports an exploration of the effect of both operative current density and plant lifetime on the cost of H_2 production. All data reported in this sheet can be calculated simply replacing I, E and plant lifetime values in the dedicated entries in the "OPEX - MW ideal comparison". Note that an important assumption was made for exploring the effect of current voltage on the hydrogen production cost: as different operative current densities would result in different system power (when keeping a constant number of cells), the size of the AEL was modified according to the operative conditions themselves as to reach a 1 MW-scale in all cases. The size was modified increasing (ideally) the number of stacks, and therefore the total number of cells in the system. It was assumed that changing the number of stacks/cells does not imply differences in the CAPEX calculation.

Further comments can be found in the Excel Spreadsheet itself, highlighted in yellow.

Fixed parameters and assumptions made throughout the TEA

In the following, all the parameters that were fixed and assumptions that were made throughout the TEA are gathered in **Tables S1-S4**. Also, the same data are reported in the annexed Excel Spreadsheet.

Supplementary Table 1. CAPEX-related parameters assumed in the TEA.

Parameters for single cell CAPEX calculation			
DEP components	Platinum[a]	48.38	$ g^{-1}
	AvCarbMGL280[a]	500	$ m^{-2}
	D1021 Nafion™ Dispersion[a]	1.53	$ g^{-1}
	SSM[b]	11.20	$ m^{-2}
	Zirfon Perl UTP 220[c]	380	$ m^{-2}
Electrode area	Lab-scale AEL	5	cm^2
	Ideal 1 MW-scale AEL	700	cm^2
System configuration	n_{Cell} (cells per stack)	200	cells
	n_{Stacks} (stacks per system)	5	stacks
Parameters for breaking down the cost of a generic 1 MW-scale AEL plant			
System CAPEX breakdown	Average stack cost (1 MW)[d]	270	$ kW^{-1}
	Stack CAPEX share[e]	45	% of total CAPEX
	BoP CAPEX share[e]	55	% of total CAPEX
System operative parameters[d]	Operative voltage	1.8	V
	Operative current density	1	A cm^{-2}
	Electrode area	700	cm^2
	Single cell power	1260	W

n_{Cell} (cells per stack)	200	cells
n_{Stacks} (stacks per system)	5	stacks
Gross system power	1.26	MW
Energy efficiency$_{HHV}$	82	%
Net system power	1.03	MW

[a] *Ref.* (Mayyas et al., 2019).

[b] *https://www.alibaba.com/product-detail/Stainless-Steel-Mesh-60-201-304_1600242671076.html?spm=a2700.7735675.normal_offer.d_image.58347f6cFNBTjP&s=p*

[c] *Price from supplier*.

[d] *Ref.* (Lee et al., 2021).

[e] *Footnote* [1].

Supplementary Table 2. OPEX-related parameters assumed in the techno-economic analysis

$OPEX_{Process\ water}$	Process water usage[a]	10	L kg^{-1}_{H2}
	Process water cost[b]	0.0014	$ L^{-1}_{H2}
Other OPEX entries[a]	$OPEX_{Labour}$	0.3	% of total CAPEX
	$OPEX_{Maintainance}$	2.5	% of total CAPEX
	$OPEX_{Ancillary}$	1	% of total CAPEX
-	Time of operation	8400	h $year^{-1}$

[a] *Ref.* (Lee et al., 2021)

[b] *Ref.* (Yates et al., 2020)

Supplementary Table 3. Financial parameters assumed in the techno-economic analysis

Interest rate	4.5	%
Plant lifetime[a]	10 – 20 – 30	years
Capital Recovery Factor	0.126	
Electricity cost[b]	0.02	\$ kWh^{-1}

[a] *Subjected to a sensitivity analysis. Average plant lifetime for MW-scale AELs is ca. 30 years.*[1]

[b] *Subjected to a sensitivity analysis. Target price of renewable electric energy.*[2]

[2] Green Hydrogen Cost Reduction – Scaling up electrolysers to meet the 1.5 °C climate goal. Int. Renew. Energy Agency (IRENA), 2020. https//irena.org/publications/2020/Dec/Green-hydrogen-cost-reduction.

Supplementary Table 4. Electrochemical and process-related parameters assumed in the techno-economic analysis

Faradaic efficiency	100	%
Number of exchanged electrons	2	$mol_{e-}\ mol^{-1}_{H2}$
Faraday's constant	96485	$C\ mol^{-1}_{e-}$
H_2 molar mass	2.016	$g\ mol^{-1}$
HHV_{H2} (hydrogen higher heating value)	141.7	$kJ\ g^{-1}_{H2}$
LHV_{H2} (hydrogen lower heating value)	120.0	$kJ\ g^{-1}_{H2}$

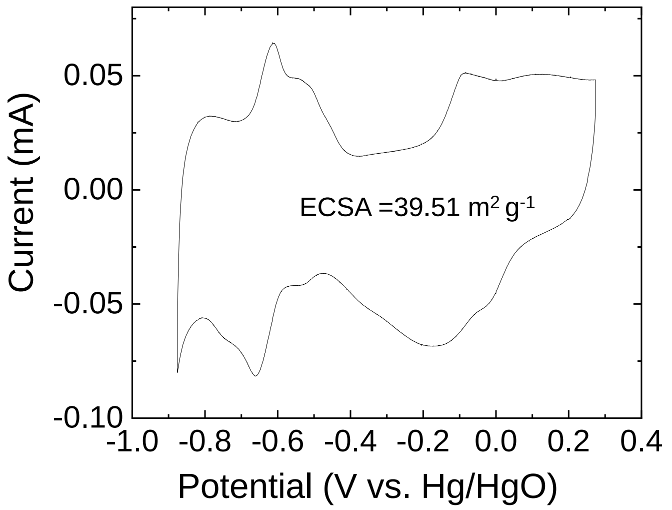

Supplementary Figure 1. a) RDE CV curve of Pt/C catalysts in 1 M KOH (50th CV scan, potential scan rate = 50 mV s^{-1}, m_{Pt} =5.3 µg). These data were used to estimate the ECSA of our Pt/C through the H_{UPD} method.

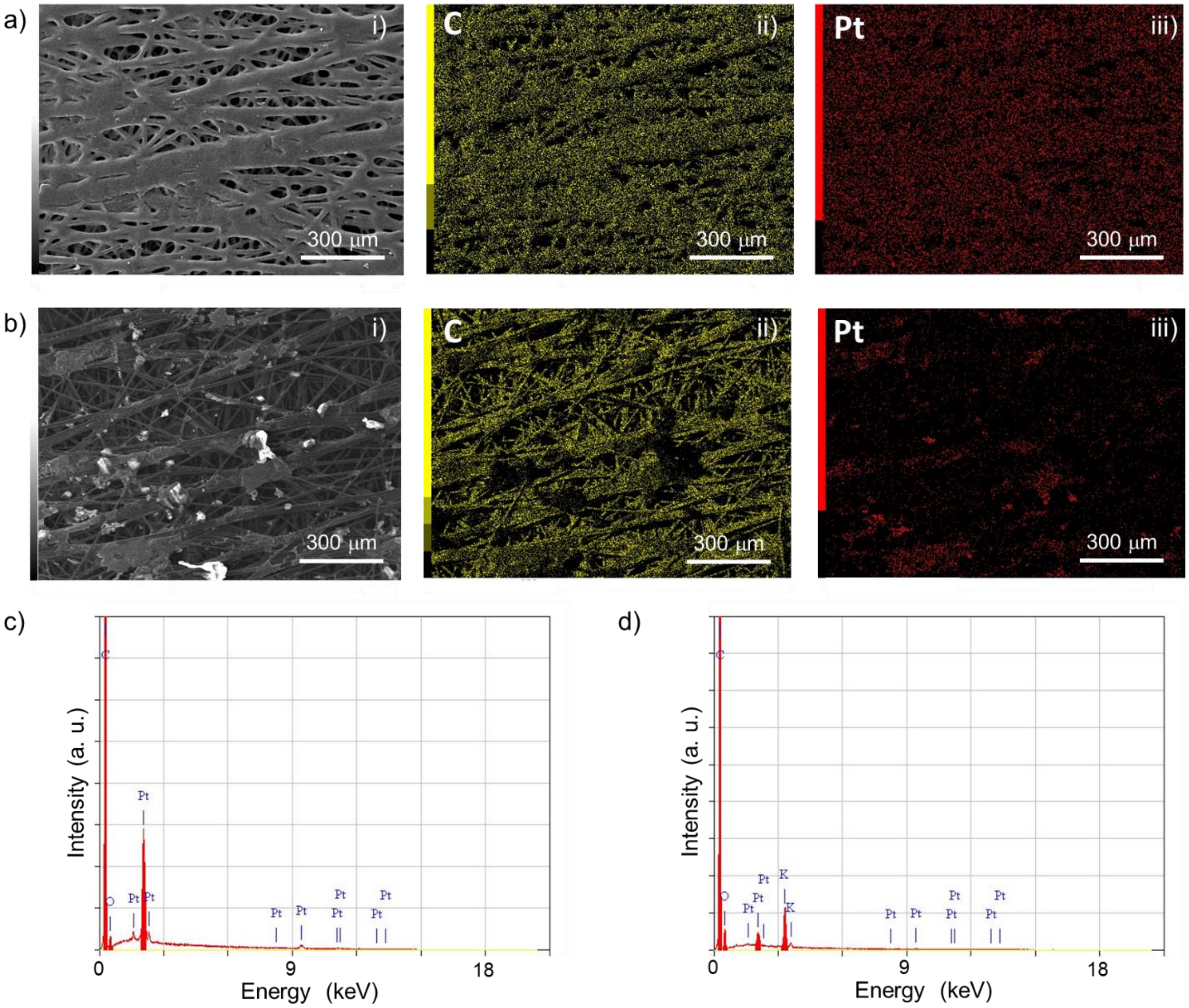

Supplementary Figure 2. a,b) SEM image (panels i) together with corresponding EDS maps for C (panels ii) and Pt (panels iii) and c,d) EDS spectra measured for the binder-free Pt/C cathode before (panels a and c) and after the AST of the corresponding AEL (panels b and d). m_{Pt} = 300 μg cm^{-2}.

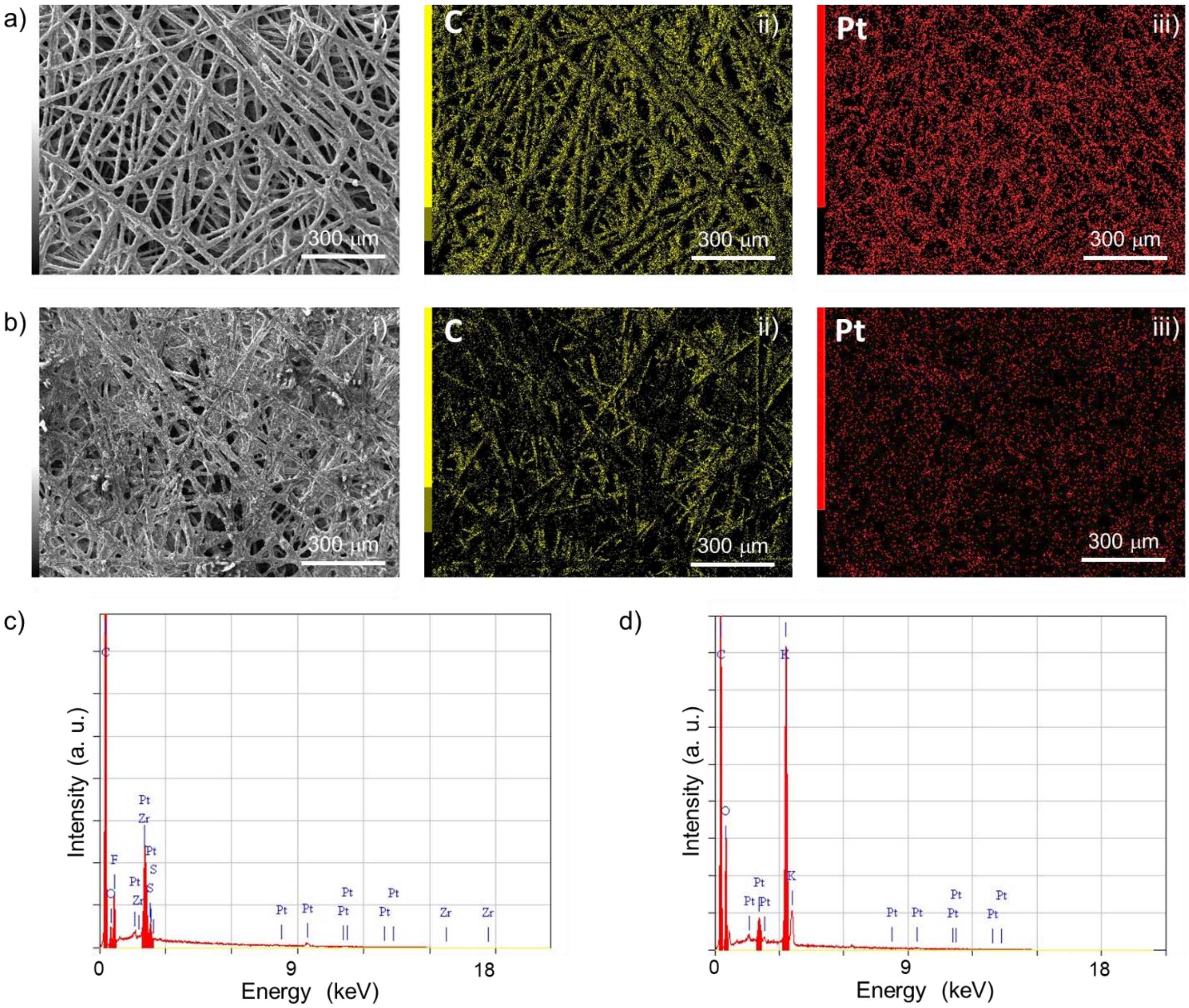

Supplementary Figure 3. a,b) SEM image (panels i) together with corresponding EDS maps for C (panels ii) and Pt (panels iii) and c,d) EDS spectra measured for the as-produced Pt/C cathode with Nafion content = 10 wt% before (panels a and c) and after the AST of the corresponding AEL (panels b and d). m_{Pt} = 300 µg cm^{-2}.

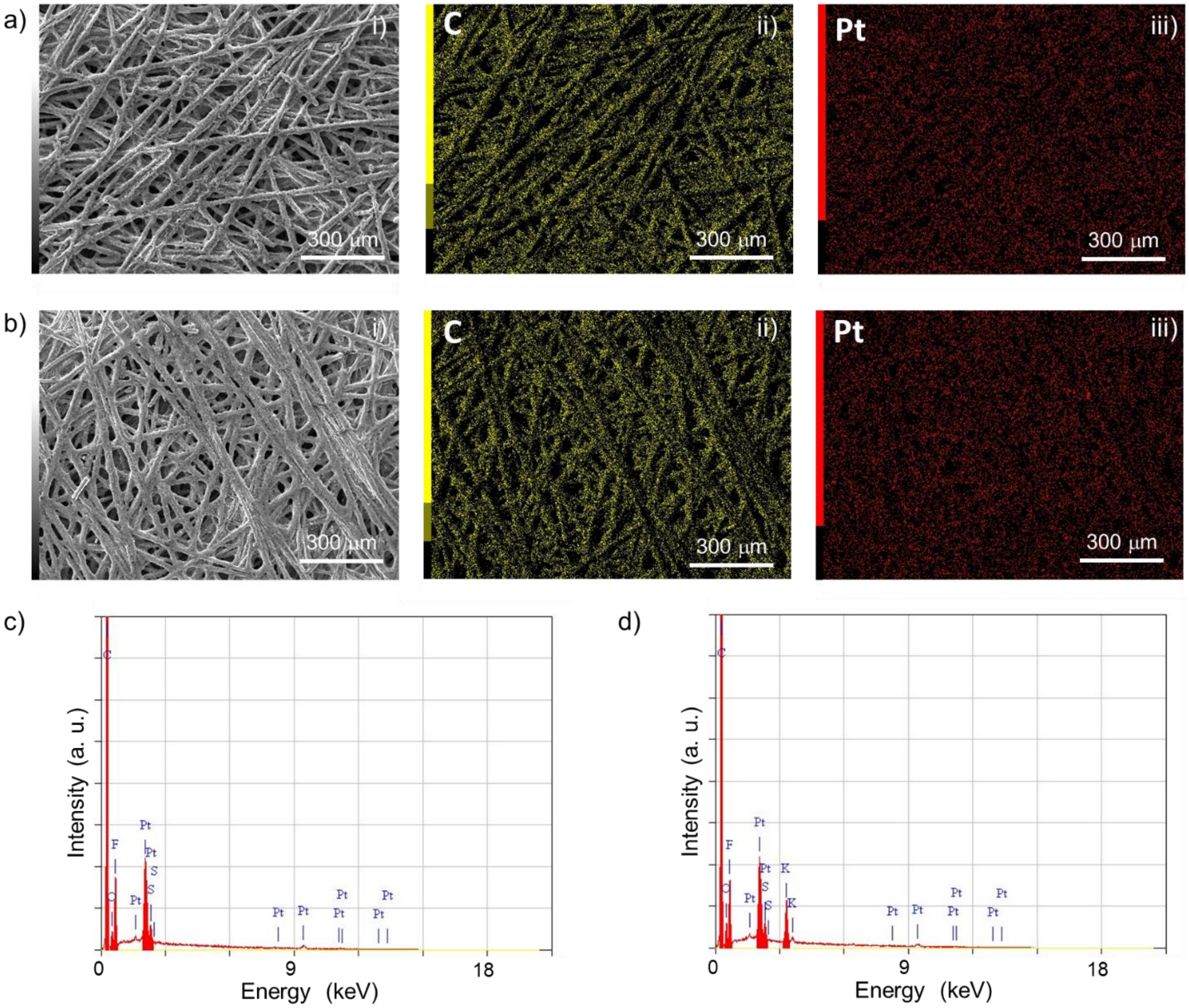

Supplementary Figure 4. a, b) SEM image (panels i) together with corresponding EDS maps for C (panels ii) and Pt (panels iii) and c,d) EDS spectra measured for the as-produced Pt/C cathode with Nafion content = 25 wt% before (panels a and c) and after the AST of the corresponding AEL (panels b and d). m_{Pt} = 300 µg cm^{-2}.

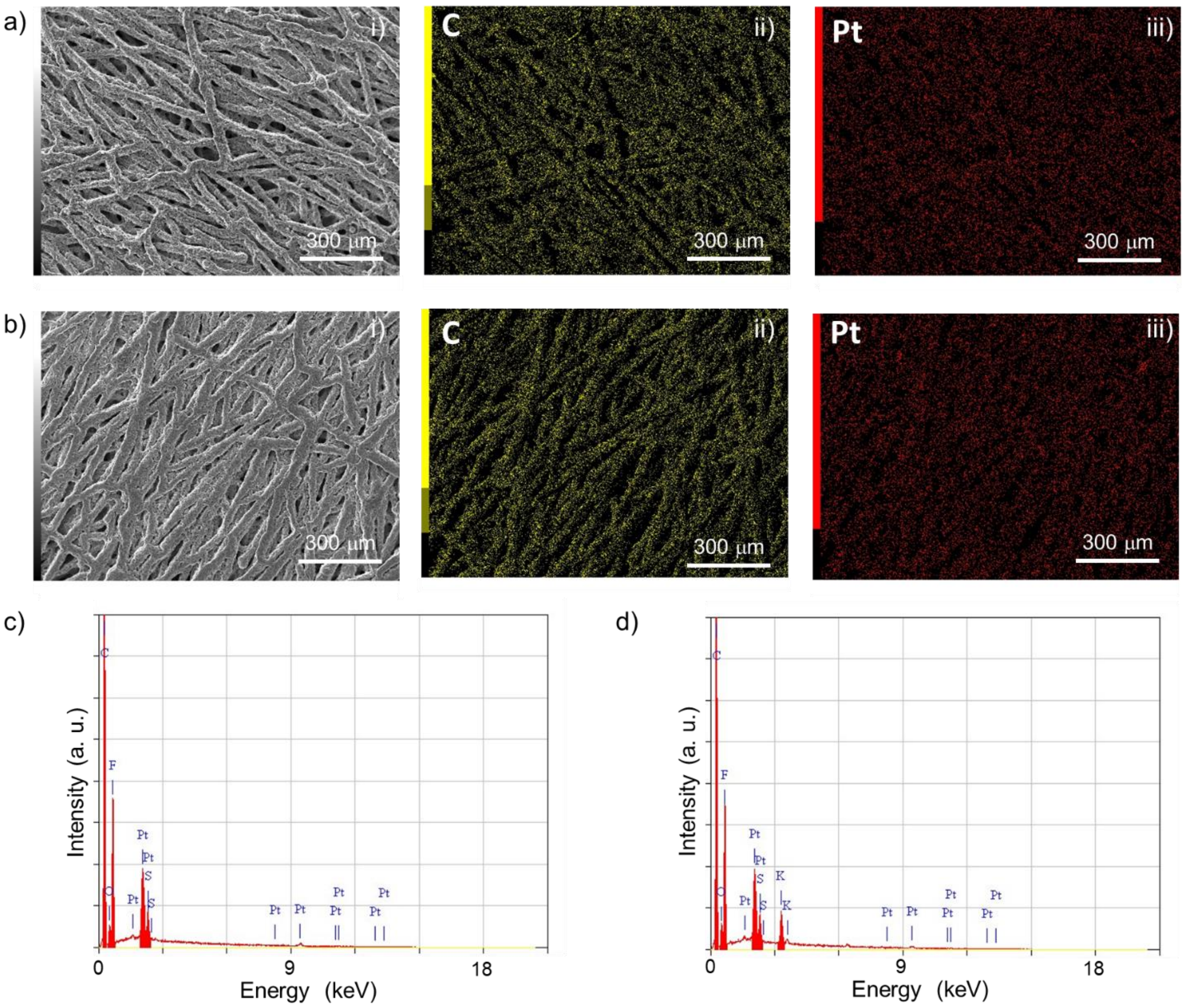

Supplementary Figure 5. a, b) SEM image (panels i) together with corresponding EDS maps for C (panels ii) and Pt (panels iii) and c,d) EDS spectra measured for the as-produced Pt/C cathode with Nafion content = 50 wt% before (panels a and c) and after the AST of the corresponding AEL (panels b and d). m_{Pt} = 300 µg cm^{-2}.

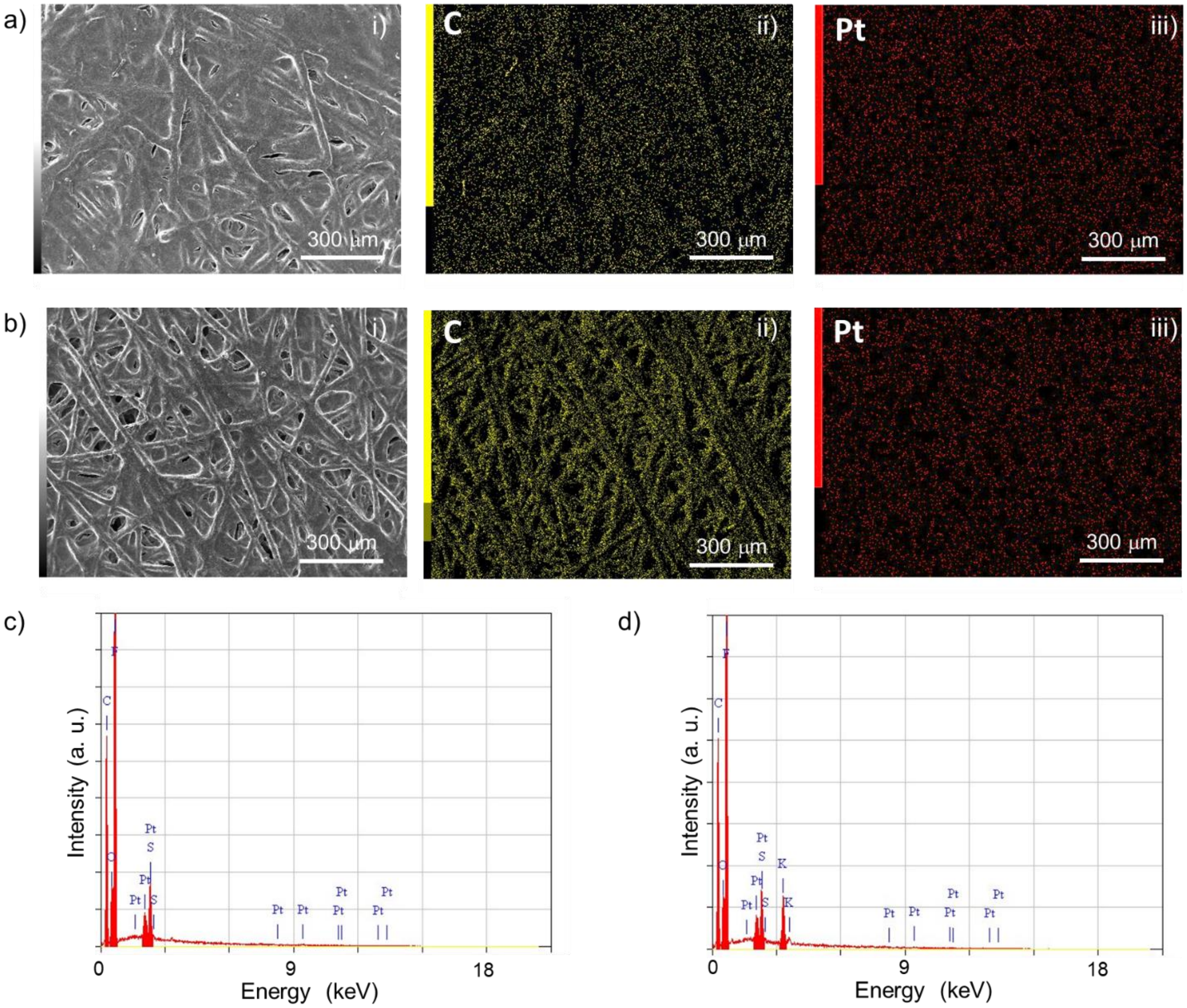

Supplementary Figure 6. a, b) SEM image (panels i) together with corresponding EDS maps for C (panels ii) and Pt (panels iii) and c,d) EDS spectra measured for the as-produced Pt/C cathode with Nafion content = 80 wt% before (panels a and c) and after the AST of the corresponding AEL (panels b and d). m_{Pt} = 300 µg cm^{-2}.

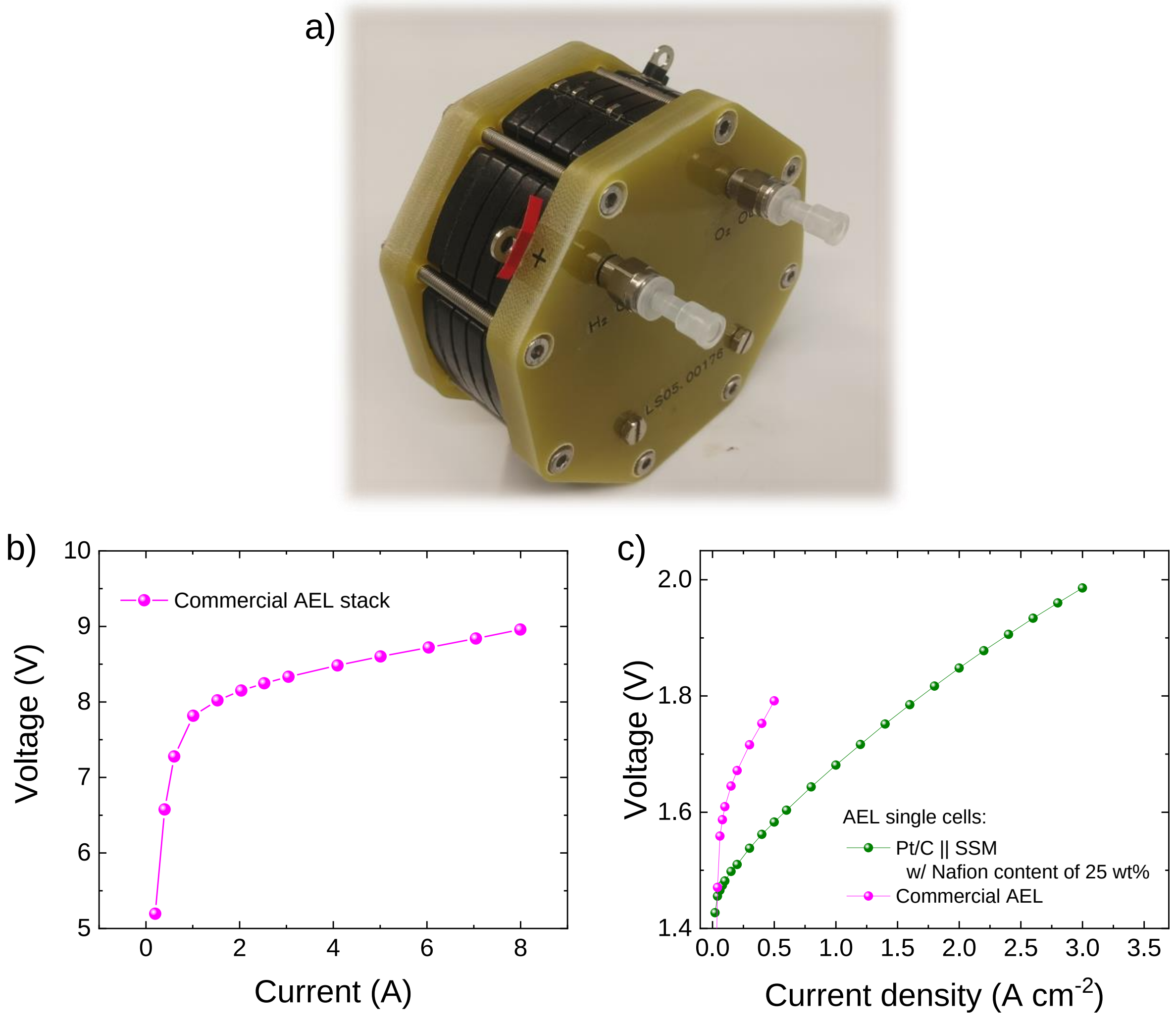

Supplementary Figure 7. a) Photograph of a commercially available AEL stack (5 single cells, electrode area = 16 cm^2) (Fuel cell store). Membrane: proprietary porous polymer; electrode material: proprietary nickel based electrode. b) Cathodic galvanostatic polarization curve measured for the commercial AEL stack. c) Comparison between the galvanostatic polarization curves measured for the commercial AEL single cell and our Pt/C || SSM with a Nafion content of 25 wt%.

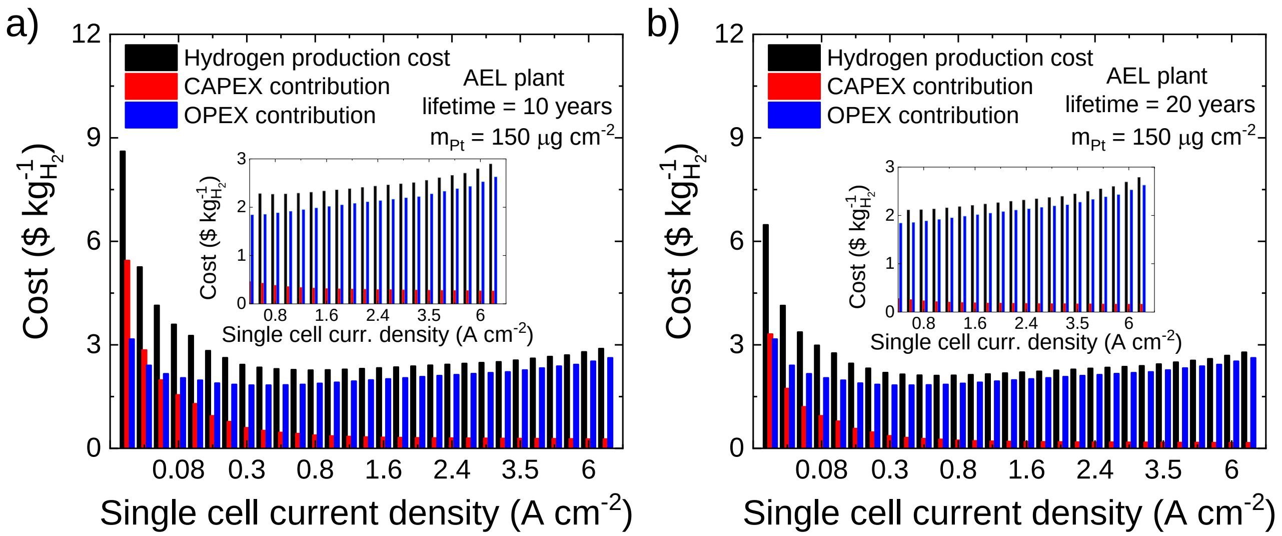

Supplementary Figure 8. OPEX, CAPEX and overall hydrogen production for 1 MW-scale AEL plant based on the Pt/C cathodes with m_{Pt} of 150 µg cm^{-2}, as a function of the single current density, for plant lifetime of a) 10 years and b) 20 years. The inset panels show the graph magnification for current densities ≥ 400 mA cm^{-2}.

Table S5. Comparison between the water splitting performances of our AELs and those of ELs reported recently

**Electrolyzer type**	**Temperature (ºC)**	**Anode (mg cm^{-2})**	**Cathode (mg cm^{-2})**	**Diaphragm/ Membrane**	**@0.5 A cm^{-2} (V)**	**@1 A cm^{-2} (V)**	**@2 A cm^{-2} (V)**	**Ref.**
**AEL**	**80 (30 wt% KOH),**	**5-stacked SS mesh**	**Pt/C w/ 25 % nafion content (0.15)**	**Zirfon Perl UPT 220**	**1.58**	**1.68**	**1.85**	**This work**
AEL	80 (30 wt% KOH)	NiFe-LDH	Raney-Ni	Zirfon Perl UPT 500	~1.75	~1.95	~2.4	*Chem. Eng. J.* **2022**, 428, 131149
	80 (30 wt% KOH)	NiFe-LDH	Raney-Ni	80 wt% ZrO_2/5 wt% CNCs	~1.62	~1.75	~1.95	
	80 (10 wt% KOH)	NiFe-LDH	Raney-Ni	80 wt% ZrO_2/5 wt% CNCs	~1.75	1.9	2.3	
AEL	80 (30 wt% KOH)	Raney-Ni	Raney-NiMo	Zirfon Perl UPT 500	/	~1.9	~2.3	*J. Electrochem. Soc.* **2016**, 163, F3197
AEL	80 (24 wt% KOH)	Raney-Ni	Raney-NiMo	Zirfon Perl UPT 500	~1.73	~1.95	~2.4	*Energy Environ. Sci.*, **2019**, 12, 3313-3318
AEL	80 (30 wt% KOH)	NiFe-LDH	Raney-Ni	300 μm-thick Z80 separator (ZrO_2 and	/	~1.8	~2.1	*J. Membrane Sci.* **2020**, 616, 118541

				PPSU-based film)				
AEL	80 (30 wt% KOH)	Ni plate	Ni plate	Zirfon Perl UPT 500	~2	/	/	*ACS Sustainable Chem. Eng.* **2018**, 6, 4829−4837
PEM	50 (0.5 M H_2SO_4)	$Ta_{0.1}Tm_{0.1}Ir_{0.8}O_{2-\delta}$ (0.2)	20 wt% Pt/C (0.2)	Nafion 117	~1.61	1.766	1.935	*Nat. Nanotechnol.* **2021**, 16, 1371–1377
PEM	80 (H_2O)	IrO_2@TiO_2 (Ir-loading: 0.4)	60 wt% Pt/C (0.5)	Nafion N212 or N115	~1.6	1.67	~1.78	*Appl. Catal. B: Environ.* **2020**, 269, 118762
PEM	80 (H_2O)	$Ir_{0.7}Ru_{0.3}O_x$ (1.8)	Pt/C (0.5)	Nafion 115	~1.54	1.66	~1.84	*Adv. Energy Mater.* **2018**, 9, 1802136
PEM	80 (H_2O)	$IrRuO_x$ (3)	Pt black (3)	Nafion 115	/	~1.67	1.84	*Nano Energy* **2018**, 47, 434–441
PEM	80 (H_2O)	Ir black (2)	FeMoS(mw) (4)	Nafion 212	1.77	1.85	/	*ACS Catal.* **2020**, 10, 14336−14348
PEM	60 (H_2O)	$Y_{1.75}Ca_{0.25}Ru_2O_7$ (4.1)	Pt/C (1.5)	Nafion 212	~1.58	~1.67	/	*Appl. Catal. B: Environ.* **2020**, 260, 118176
PEM	60 (H_2O)	IrO_2 (3)	Pt/C (1.5)	Nafion 212	~1.64	~1.75	/	
PEM	38 (H_2O)	Ir	Pt	Nafion 212	~1.7	~1.8	1.97	*Energy Environ. Sci.* **2017**, 10, 2521-2533

AEM	60 (1M KOH)	Ni_2P @ $FePO_xH_y$	$MoNi_4/MoO_2$	Sustainion X37-50	~1.75	1.84	/	*Appl. Catal. B: Environ.* **2022**, 306, 121127
AEM	90 (6M KOH)	IrO_2 (1.5)	Pt/C (1.5)	NPBI^ ion-solvating membrane	~1.57	~1.69	~1.9	*J. Membrane Sci.* **2022,** 643, 120042
AEM	60 (H_2O)	IrO_2 (~4)	PtRu/C (Pt, Ru loading: 2,2)	HTMA-DAPP*(~50 μ m)	~1.65	1.79	/	*ACS Appl. Mater. Interfaces* **2021,** 13, 50957−50964
AEM	90 (H_2O)	fluoride-incorporated Fe_xNi_yOOH on Ni foam (4.8)	47% Pt/C (0.94 mg Pt)	poly(aryl piperidinium) hydroxide exchange membrane (PAP-TP-85, 20 μm)	~1.65	~1.8	/	*ACS Catal.* **2021**, 11, 264−270
AEM	50 (H_2O)	NiCoOx:Fe (3)	Pt black (3)	FAA-3 (FumaTech)	~2.1	~2.45	/	*ACS Catal.* **2019,** 9, 7–15,
AEM	50 (0.3 M KOH)	IrO_x (3.0 ± 0.1)	PtNi (3.0 ± 0.1)	XION™ Composite-72–10CL-30 μm	/	1.79	/	*Electrochim. Acta* **2022,** 409, 140001
AEM	50 (1 M KOH)	$Ni(OH)_2$-Fe (4)	Raney NiMo (25.2)	Tokuyama A201 (28 μm)	~1.7	~1.85	2.046	*ACS Appl. Energy Mater.* **2022,** 5, 2221–2230

AEM	42-45 (1M KOH)	$Ni_{0.75}Fe_{2.25}O_4$	Pt/C (1)	Sustainion X37-50	~1.65	1.75	1.9	*Chem. Eng. J.* **2021,** 420, 127670
AEM	42-45 (1M KOH)	IrO_2 (4)	Pt/C (1)	Sustainion X37-50	~1.67	1.84	/	
AEM	50 (1M KOH)	NiFeV LDH (3.95)	Pt/C	Sustainion X37-50	~1.56	~1.65	~1.79	*Small* **2021,** 17, 2100639
AEM	30 (0.1M KOH)	$Cu_{0.81}Co_{2.19}O_4$	40 wt% Pt/C (1)	Fumasep FAA-3-PE-30	~1.87	/	/	*ACS Appl. Mater. Interfaces* **2018,** 10, 38663-38668
AEM	45 (1M KOH)	IrO_x (4)	40 wt% Pt/C (1)	Sustainion X37-50	/	~1.75	~1.87	*Appl. Catal. B: Environ.* **2020,** 278, 119276
AEM	50 (1M KOH)	$Cu_{0.5}Co_{2.5}O_4$ (10)	40 wt% Pt/C (1)	Sustainion X37-50	~1.65	~1.74	/	*J. Mater. Chem. A* **2020,** 8, 4290–4299
AEM	80 (1M KOH)	NiFe-LDH (2.5)	Pt/C (1.3)	Sustainion X37-50	~1.52	1.59	/	*ACS Catal.* **2020,** 10, 1886-1893
AEM	60 (1M KOH)	IrO_x (1.9)	Pt/C (1.2)	Sustainion X37-50	~1.55	1.67	/	
AEM	70 (1 M KOH)	60 wt.% PtRu/C (0.4 mg Pt-Ru metal)	60 wt.% Pt/C (0.4 mg Pt)	AEM based on quaternised poly-carbazole (QPC-TMA) (50 μm)	~1.55	~1.62	~1.74	*Energy Environ. Sci.*, **2020,** 13, 3633-3645

AEM	20 (1 M KOH)	Fe-NiMo-NH_3/H_2 (3)	NiMo-NH_3/H_2 (3)	Sustainion X37-50	~1.66	1.77	/	*Adv. Energy Mater.* **2020,** 10, 2002285
AEM	80 (1 M KOH)	Fe-NiMo-NH_3/H_2 (3)	NiMo-NH_3/H_2 (3)	Sustainion X37-50	1.52	1.57 V	/	
AEM	60 (1 M KOH)	$NiFe_2O_4$ (2)	NiFeCo (2)	Sustainion 37-50	~1.8	1.9	/	*Int. J. Hydrogen Energy* **2017,** 42, 29661–29665

Note: *The cell voltage values with the symbol (~) are extracted from their corresponding polarization plots.*

References

Lee, B., Cho, H. S., Kim, H., Lim, D., Cho, W., Kim, C. H., et al. (2021). Integrative techno-economic and environmental assessment for green H2 production by alkaline water electrolysis based on experimental data. *J. Environ. Chem. Eng.* 9, 106349. doi: 10.1016/j.jece.2021.106349.

Mayyas, A., Ruth, M., Pivovar, B., Bender, G., and Wipke, K. (2019). Manufacturing Cost Analysis for Proton Exchange Membrane Water Electrolyzers. Available at: https://www.nrel.gov/docs/fy19osti/72740.pdf.

Yates, J., Daiyan, R., Patterson, R., Egan, R., Amal, R., Ho-Baille, A., et al. (2020). Techno-economic Analysis of Hydrogen Electrolysis from Off-Grid Stand-Alone Photovoltaics Incorporating Uncertainty Analysis. *Cell Reports Phys. Sci.* 1, 100209. doi: https://doi.org/10.1016/j.xcrp.2020.100209.
